# Apheresis for the treatment of relapses in MS and NMOSD: reduced antibody reactivities, gene expression changes and potential clinical response indicators

**DOI:** 10.3389/fimmu.2025.1531447

**Published:** 2025-01-30

**Authors:** Michael Hecker, Brit Fitzner, Isis Ludwig-Portugall, Friederike Bohne, Edmar Heyland, Juliane Klehmet, Matthias Grothe, Matthias Schwab, Alexander Winkelmann, Stefanie Meister, Ales Dudesek, Hannah Wurm, Ilya Ayzenberg, Ingo Kleiter, Corinna Trebst, Martin W. Hümmert, Bernhard Neumann, Klaus Eulitz, Dirk Koczan, Uwe K. Zettl

**Affiliations:** ^1^ Division of Neuroimmunology, Department of Neurology, Rostock University Medical Center, Rostock, Germany; ^2^ R&D Apheresis, Miltenyi Biotec B.V. & Co. KG, Teterow, Germany; ^3^ Center for Multiple Sclerosis, Department of Neurology, Jüdisches Krankenhaus Berlin, Berlin, Germany; ^4^ Department of Neurology, University Medicine Greifswald, Greifswald, Germany; ^5^ Department of Neurology, Jena University Hospital, Jena, Germany; ^6^ Department of Neurology, St. Josef-Hospital, Ruhr University Bochum, Bochum, Germany; ^7^ Marianne-Strauß-Klinik, Behandlungszentrum Kempfenhausen für Multiple Sklerose Kranke gGmbH, Berg, Germany; ^8^ Department of Neurology, Hannover Medical School, Hannover, Germany; ^9^ Department of Neurology, University of Regensburg, Bezirksklinikum, Regensburg, Germany; ^10^ Department of Neurology, Donau-Isar-Klinikum Deggendorf, Deggendorf, Germany; ^11^ Institute of Immunology, Rostock University Medical Center, Rostock, Germany

**Keywords:** multiple sclerosis, neuromyelitis optica spectrum disorder, acute relapse, apheresis, glucocorticoids, antibodies, lymphocytes, gene expression

## Abstract

**Background:**

High-dose glucocorticoids are the standard treatment for acute relapses in patients with multiple sclerosis (MS) or neuromyelitis optica spectrum disorder (NMOSD). Therapeutic apheresis can be considered for the escalation of relapse therapy, but some patients still do not recover sufficiently. We aimed to explore the effects of apheresis on humoral and cellular immune parameters and to identify features that correlate with beneficial clinical outcomes.

**Methods:**

We studied two cohorts comprising a total of 63 patients with MS or NMOSD who were undergoing relapse therapy with either methylprednisolone or apheresis. Blood samples were collected immediately before and after therapy to isolate plasma or serum as well as immune cells. We then measured (1) concentrations of the immunoglobulin isotypes IgG, IgM and IgA, (2) antibody reactivities against 12 peptides derived from potential autoantigens and Epstein-Barr virus proteins, (3) frequencies of CD19^+^ B cells, CD3^+^ T cells and CD14^+^ monocytes, (4) transcriptome profiles of CD19^+^ B cells and CD4^+^ T cells and (5) mRNA levels of 7 cytotoxicity-related genes in CD4^+^ T cells. The data were compared with regard to changes under therapy and with regard to differences between clinical responders and non-responders.

**Results:**

The initial therapy with methylprednisolone had no significant effect on immunoglobulin levels and (auto)antibody reactivities (*n*_max_=27 MS patients). In contrast, MS patients who underwent apheresis (*n*_max_=27) showed strong immunoglobulin reduction rates, especially for IgG, and decreased antibody reactivities against all tested peptides. EBNA1 (amino acids 391-410) was the only peptide that also reached the significance level in NMOSD patients (*n*=9). Non-responders to apheresis (*n*=12) had on average higher anti-EBNA1 (391-410) reactivities than responders (*n*=24) at baseline. Apheresis also led to a decrease in the proportion of monocytes, an increase in the proportion of T cells (*n*=29 patients with MS or NMOSD) and moderate transcriptome changes (*n*_max_=4 MS patients). A gene expression signature that is characteristic of CD4^+^ cytotoxic T lymphocytes (CD4-CTLs) was found to be elevated at baseline in non-responders to apheresis, although this could not be validated with statistical significance (*n*=19 MS patients).

**Conclusion:**

Our data reveal that therapeutic apheresis in MS rapidly leads to a significant decrease in IgG reactivities against EBNA1 (391-410) and cross-reactive targets such as GlialCAM (370-389) and also has an impact on the gene expression of B cells and T cells. Further studies are required to verify whether anti-EBNA1 (391-410) antibody reactivities and the expression of CD4-CTL-related genes may be indicative of the individual clinical response to this therapy.

## Highlights

- Therapeutic apheresis is an option for the treatment of steroid-refractory relapses in patients with neurological diseases, but individual clinical outcomes are difficult to predict.
- Our data demonstrate that apheresis leads to markedly reduced (auto)antibody reactivities and a differential expression of genes in both B cells and CD4^+^ T cells.
- Non-responders showed higher IgG reactivities against EBNA1 (391-410) and a higher expression of cytotoxicity-related genes in CD4^+^ T cells before apheresis.

## 1 Introduction

Multiple sclerosis (MS) and neuromyelitis optica spectrum disorder (NMOSD) are chronic immune-mediated diseases that affect the central nervous system (CNS) (1). The global prevalence of MS is 35.9 per 100000 people (2), whereas NMOSD is rarer, affecting about 0.5-5 per 100000 people (3). Both diseases are characterized by focal and diffuse inflammation, demyelination and neuro-axonal degeneration in the brain and spinal cord (4, 5). However, some lesions are more typical for MS (e.g., periventricular and juxtacortical lesions), while others are more common in NMOSD (e.g., optic nerve and longitudinally extensive spinal cord lesions) (6). The two conditions exhibit a wide range of symptoms, including visual disturbances, motor and sensory deficits as well as cognitive impairment (3, 7, 8). In 85-90% of the patients with MS, the disease starts with a single clinical event, called clinically isolated syndrome (CIS), continues as relapsing-remitting MS (RRMS) after fulfilling the diagnostic criteria and later transitions to secondary progressive MS (SPMS) with gradual worsening of neurologic disability (9). Similarly, NMOSD has a relapsing course in more than 90% of cases (5). The pathomechanisms of MS involve complex interactions between B cells, T cells and microglia as well as the production of antibodies (10). No MS-specific autoantibody pattern has been defined, but antibody reactivities against peptide sequences of a broad range of CNS antigens and other human proteins have been reported for minor subgroups of MS patients (11–18). NMOSD, on the other hand, is associated with the presence of anti-AQP4 antibodies that target and damage astrocytes in ~80% of cases (5). Genetic, environmental and lifestyle factors contribute to risk and severity of MS and NMOSD (19, 20). However, some established risk factors for MS, such as infection with Epstein-Barr virus (EBV) (21, 22), do not appear to be (as strongly) associated with susceptibility to NMOSD (23, 24). The aims of disease-modifying therapies (DMTs) or long-term immunotherapies (e.g., with monoclonal antibody drugs) for MS and NMOSD are to reduce the frequency of relapses and to slow disease progression (25-27), while acute treatments focus on managing relapses to speed up recovery.

Relapses are defined as new symptoms or worsening of preexisting neurologic symptoms lasting longer than 24 h at least 30 days after the most recent relapse in the absence of fever or infection (28). Acute relapses are typically associated with an objective worsening of disability (3, 29), and therefore effective relapse prevention and relapse treatment are crucial for maintaining patients’ neurological function and quality of life. The recommended treatment for a relapse is the use of high-dose short-term glucocorticoids (GCs), usually by intravenous or oral administration of methylprednisolone at a dose of 500-1000 mg per day for 3-5 days (30). For the escalation of relapse therapy, a second course of high-dose GC treatment with up to 2000 mg methylprednisolone daily for 3-5 days can be considered within ~2 weeks (31, 32). In the case of steroid-refractory relapses, an escalating therapy with apheresis by means of plasma exchange or immunoadsorption may be indicated (32–34). However, some patients do not recover completely despite intense relapse therapy. Predictors of incomplete functional recovery include older patient age, higher relapse severity, polysymptomatic presentation and longer time from relapse onset to start of therapy (35–39). Moreover, compared with MS, NMOSD relapses tend to be more severe and less responsive to GC treatment (31, 40).

Various studies have been conducted to achieve a better understanding of the cellular and molecular effects of relapse therapy and to infer biomarkers that are predictive of individual therapeutic outcomes. GCs were shown to cause a short-term reduction in the number of T cells in the blood, whereas the number of B cells is less affected (41). In a recent transcriptome study, we have identified genes that were significantly up- or downregulated in CD19^+^ B cells and CD4^+^ T cells after relapse therapy with high-dose methylprednisolone (42). However, genes that were previously suggested as potential prognostic biomarkers of the clinical response to GC therapy could not be confirmed in our data (42). Therapeutic apheresis has been demonstrated to substantially reduce the levels of immunoglobulins (Igs) (43–45) and to decrease the frequency of B-cell subsets in the peripheral blood (45, 46). The response to apheresis treatment is presumably related to immunopathological patterns, which can be determined through histological examination of a brain biopsy, though this is not routinely performed (47, 48). There is also evidence that a higher frequency of circulating IFNG^+^ T helper 1 cells before apheresis may correlate with a beneficial clinical response (46). However, this finding has yet to be confirmed. To our knowledge, there is no study to date in which the transcriptome profile of blood cells from patients before and after apheresis has been compared. In addition, the effect of therapeutic apheresis on (auto)antibody repertoires has not been explicitly investigated in patients with MS.

In the present study, we analyzed blood samples that were obtained from patients with MS and patients with NMOSD before and after the treatment of an acute relapse. We determined the extent to which therapeutic apheresis is associated with (1) changes in the binding of antibodies to selected peptides, (2) shifts in the proportions of basic immune cell populations and (3) transcriptome dynamics in B cells and CD4^+^ T cells. Based on these data, we further examined whether the patients’ clinical response to apheresis is associated with (auto)antibody reactivities, cell-type compositions or gene expression levels. Our work therefore explores the mechanisms of action of the therapy and assesses possible clinical response indicators.

## 2 Methods

### 2.1 Study cohorts

**Figure 1:**
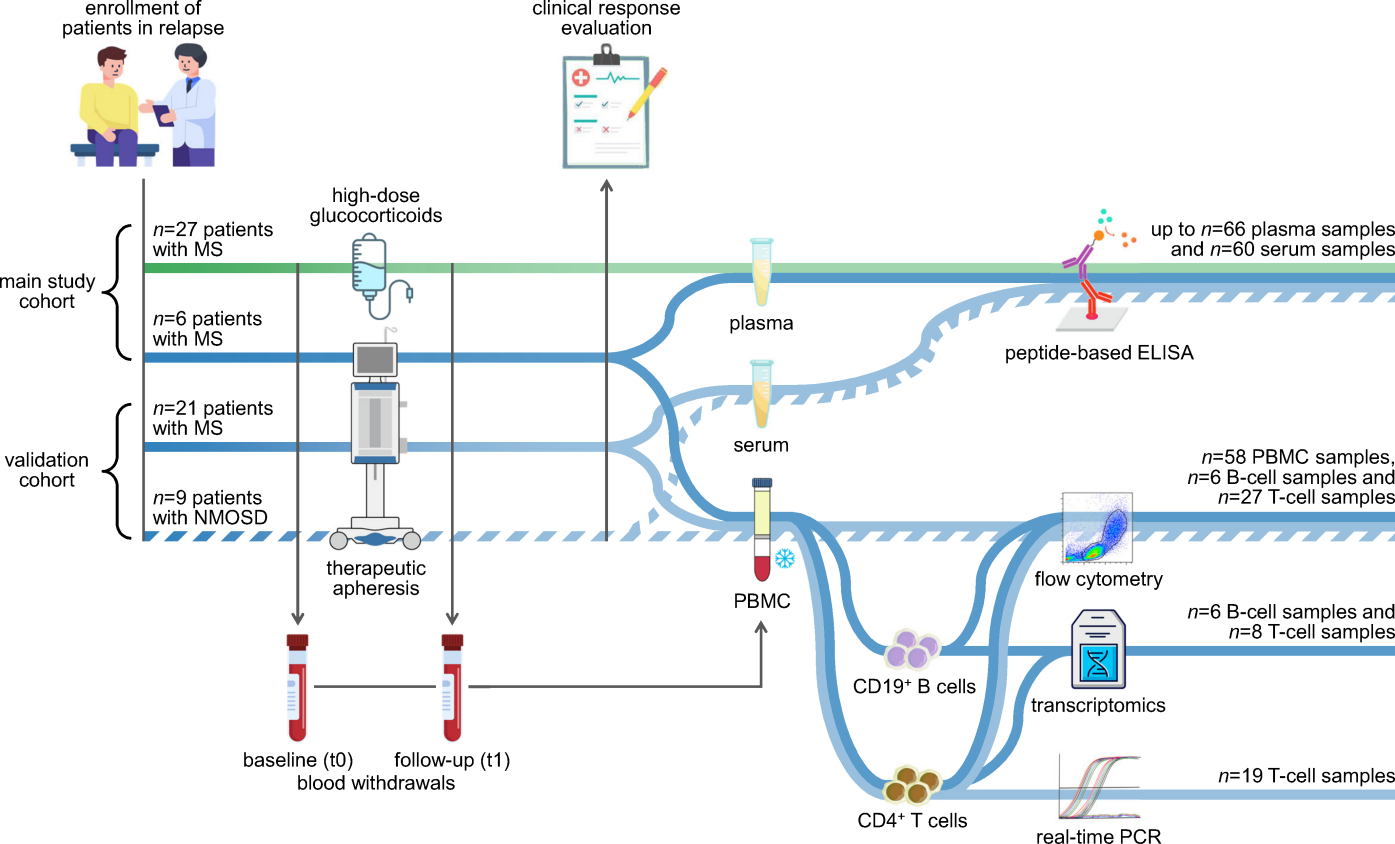
Study design and experimental workflow. The analyses were based on two independent cohorts: the main study cohort (*n*=33 patients) and the validation cohort (*n*=30 patients). Patients with MS (solid lines) and patients with NMOSD (broken line) were recruited at the time of an acute relapse. The patients received either high-dose methylprednisolone (green line) or therapeutic apheresis (blue lines). Peripheral blood was taken immediately before and after the relapse treatment. The blood samples were used to separate plasma or serum as well as PBMC. The plasma and serum samples were used to measure antibody reactivities against potential linear epitopes by indirect ELISAs. The PBMC samples from the validation cohort were cryopreserved and later used for a basic immunophenotyping. CD19^+^ B cells and CD4^+^ T cells were isolated from a subset of PBMC samples, and their purity was assessed by flow cytometry. RNA from the isolated cells was employed for a transcriptome profiling using Clariom D arrays (main study) and for real-time PCR measurements (validation). The data were analyzed to explore changes in the humoral and cellular immune signature following apheresis and to identify markers of the patients’ clinical response to relapse therapy. We refer to our previous study regarding transcriptome data of MS patients receiving methylprednisolone (42). ELISA, enzyme-linked immunosorbent assay; MS, multiple sclerosis; NMOSD, neuromyelitis optica spectrum disorder; PBMC, peripheral blood mononuclear cells; PCR, polymerase chain reaction.

This study was based on two patient cohorts: a main study cohort and a validation cohort (**Figure 1**). In the main study, MS patients in relapse were recruited at 4 study centers in Germany (Berlin, Greifswald, Jena and Rostock). The validation cohort was enrolled at 3 study centers in Germany (Bochum, Hannover and Regensburg) and comprised patients with MS or NMOSD who also had a relapse. The MS patients had been diagnosed with either CIS/RRMS or SPMS with superimposed relapses according to the revised McDonald criteria (9, 49). The patients with NMOSD were diagnosed according to the criteria of the International Panel for NMO Diagnosis (50). The NMOSD patients were included in this study alongside the MS patients to investigate whether relapse therapy induces similar effects in both diseases. Only patients over the age of 18 were included in the prospective studies, and patients with substantial cognitive deficits that would hamper their study participation were excluded. Sociodemographic, clinical and medication data of the patients (e.g., age, sex, disease duration and relapse presentation) were gathered through questionnaires to be completed by the treating physicians. However, different sets of parameters were recorded for the two cohorts.

Written informed consent was obtained from all patients in advance. The studies were performed in compliance with the Good Clinical Practice guidelines, the Declaration of Helsinki and the European General Data Protection Regulation. Ethical approvals were obtained from the ethics committees of the University of Rostock (approval numbers A 2019-0047 and A 2020-0048) and the Ruhr University Bochum (approval number AZ 15-5395).

### 2.2 Relapse therapy and clinical response evaluation

The patients were treated and monitored following the guidelines and recommendations of the German Society of Neurology (32). Routine medical care was therefore provided to all patients. The patients either received GCs or underwent apheresis to support relapse recovery. High-dose methylprednisolone was administered as initiation treatment with up to 1000 mg daily for up to 5 days (main study cohort). Therapeutic apheresis (i.e., plasma exchange or immunoadsorption) was performed on several consecutive days, typically as escalation treatment for steroid-refractory relapses after one or two previous GC treatment courses (both cohorts).

To classify clinical responders and non-responders to relapse therapy, the patients were examined at baseline (i.e., prior to initiation or escalation treatment) and at a later date, usually within 4 months. The degree of neurological disability was rated using the Expanded Disability Status Scale (EDSS) (51). In the main study, the treating physicians were asked to judge whether or not an overall symptom improvement could be observed. In the validation cohort, the clinical outcome of therapeutic apheresis was evaluated slightly differently: In patients with optic neuritis, the neurological examination included a high-contrast visual acuity testing, and treatment response was defined as an improvement in the EDSS score of ≥1.0 or in visual acuity of ≥20%.

### 2.3 Blood collection and processing

In the main study, up to ~30 mL peripheral blood was collected immediately before and after relapse therapy in 8 mL BD Vacutainer Cell Preparation Tubes containing the anticoagulant sodium citrate and a blood separation medium consisting of a polyester gel and a Ficoll-Hypaque solution. The blood collection tubes were centrifuged at the respective study center and then sent to the laboratory in Rostock within 24 hours. There, plasma and peripheral blood mononuclear cells (PBMC) were separated according to the manufacturer’s instructions. The plasma samples were stored at -80°C until further use.

The blood samples of the validation cohort were taken in a similar manner before and after therapeutic apheresis. However, different blood collection tubes were used. Serum tubes were centrifuged shortly after blood withdrawal. The sera were then transferred into separate tubes and stored at -80°C. Tubes containing ethylenediaminetetraacetic acid (EDTA) were used to extract PBMC by density gradient centrifugation using Lymphoprep (Carl Roth). The PBMC samples were cryopreserved in CTL-Cryo ABC medium (Immunospot) in Bochum and shipped in liquid nitrogen to Rostock for the analyses of the present study.

### 2.4 Measurement of antibody reactivities

The concentrations of the Ig isotypes IgG, IgM and IgA were determined in the plasma samples using a Beckman Coulter AU480 analyzer. Antibody reactivities against selected linear epitopes of proteins from human and EBV were measured in plasma and serum by indirect peptide-based enzyme-linked immunosorbent assays (ELISAs).

The peptide sequences of interest (*n*=12) were previously described as (auto)antigenic in the literature (12-15, 22, 52, 53) and are listed in **Supplementary Table 1**. They were synthesized by the company Miltenyi Biotec with N-terminal acetylation and C-terminal amidation as well as a lysine linker near the N-terminus for the conjugation of biotin to the peptide sequence.

For the ELISAs with plasma samples, 1 µg/mL streptavidin (Merck) was coated on 96-well Immuno Plates (Thermo Scientific) overnight at 4°C. The plates were washed 3 times with phosphate-buffered saline (PBS)/1% Tween 20 and blocked with PBS/1% bovine serum albumin (BSA) buffer at room temperature (RT) for 1 h. Afterwards, 2 µg/mL biotinylated peptide was added and incubated at RT for 1 h. Then, the plates were washed again 3 times. To assess unspecific binding of plasma components, an uncoated control well without streptavidin and peptide was reserved for each sample. Diluted plasma samples (1:200 in blocking buffer) were added and incubated at RT for 2 h on the plates. After washing, a goat anti-human IgG (H+L) horseradish peroxidase-conjugated antibody (Invitrogen, stock concentration: 0.8 mg/mL) was diluted 1:10000, added and incubated for 1 h at RT. After washing 3 times with PBS/1% Tween 20, 100 μL of TMB substrate (SERVA Electrophoresis) was added to each well. After reacting at RT in the dark for 5 min, 100 μL stop solution (1 M H_2_SO_4_) was added per well. The optical densities (ODs) were measured at 450 nm using an Agilent BioTek Epoch Microplate Spectrophotometer. The average of the measured OD values per sample (doublets or triplets) was normalized by subtracting the mean OD value of blank wells and the OD value of the respective uncoated control. For the statistical analysis, negative values in the data were truncated to zero.

For the serum samples of the validation cohort, the ELISAs were performed with the peptide coating kit from Takara Bio. In 96-well reaction plates, 4 µg/mL of peptides in reaction buffer were coated with coupling reagent for 2 h at 37 °C. The coated wells were blocked for 1 h. The remaining steps were as described above, with the exception of using distilled water as the wash buffer.

### 2.5 Quantification of immune cell subsets

For a basic immunophenotyping by flow cytometry, PBMC samples of the validation cohort were thawed at 37°C in a water bath and washed with CTL Anti-Aggregate Wash Supplement (ImmunoSpot). The cells were then washed with PBS and stained with 0.3 µg BD Horizon Fixable Viability Stain 700 per 1×10^6^-1×10^7^ cells in PBS at RT in the dark for live/dead discrimination. After washing, the cells were resuspended in PBS containing 2 mM EDTA and 0.5% BSA, and Fc receptors were blocked with human Fc receptor Blocking Reagent (Miltenyi Biotec) according to the manufacturer’s instructions. Fluorophore-conjugated mouse anti-human antibodies against cell surface markers (CD19-PE/Cy7, CD3-APC-H7 and CD14-BV605, all from BD) were premixed in Brilliant Stain Buffer (BD) at recommended concentrations. The cells were stained at RT in the dark for 30 min. After washing the cells, data were recorded using a BD FACSAria IIIu and the BD FACSDiva software (version 8.0.2) and further processed using FlowJo (version 10.7.1) from BD. The data were automatically cleaned for technical signal anomalies using the flowAI plugin (54). Only live single cells were considered for determining the relative proportions of B cells, T cells and monocytes in PBMC.

### 2.6 Isolation of B cells and T cells

Selected fresh PBMC samples (main study cohort) and frozen-thawed PBMC samples (validation cohort) were first washed with autoMACS Running Buffer (Miltenyi Biotec). Afterwards, B cells were positively selected from the PBMC using CD19 MicroBeads and LS Separation Columns (Miltenyi Biotec). CD4^+^ T cells were obtained from the negative fraction of the B-cell separation by removing non-T cells using the Pan T Cell Isolation Kit (Miltenyi Biotec) and isolating CD4^+^ T cells using CD4 MicroBeads (Miltenyi Biotec). The purity of the isolated cells was assessed by staining a small subset of the cells with CD3-PerCP and CD4-FITC or CD20-PE antibodies (Miltenyi Biotec). The flow cytometry analysis was carried out using a FACSCalibur instrument (BD) and Flowing Software version 2.5.1 (Turku Bioscience). The remaining cells were lysed with QIAzol Lysis Reagent (Qiagen) and stored at -20°C until further use.

### 2.7 Transcriptome profiling

The RNA from CD19^+^ B cells and CD4^+^ T cells was isolated using the miRNeasy Mini Kit and the RNase-Free DNase Set (Qiagen). RNA concentrations were measured using a NanoDrop ND-1000 Spectrophotometer (Thermo Fisher Scientific), and RNA integrity was assessed with an Agilent 2100 Bioanalyzer using RNA 6000 Pico kits (Agilent Technologies). We used 100 ng of total RNA from each sample to generate amplified, fragmented and biotinylated single-stranded sense strand DNA using the GeneChip WT PLUS Reagent Kit (Applied Biosystems). Poly-A RNA controls were used to monitor the target labeling process, and hybridization controls were spiked into the hybridization cocktail to evaluate sample hybridization efficiency. The hybridization on high-resolution Clariom D arrays for human (Applied Biosystems) was carried out for 16 h at 45°C in a GeneChip Hybridization Oven 645 (Affymetrix) following the manufacturer’s instructions. After washing and staining in a GeneChip Fluidics Station 450 (Affymetrix), the microarrays were scanned using a GeneChip Scanner 3000 7G (Affymetrix).

The Affymetrix GeneChip Command Console version 4.0 was used to extract the signal intensities for the >6.7 million 25mer oligonucleotide probes per array. The Transcriptome Analysis Console (TAC) version 4.0.3 (Applied Biosystems) was then used for processing the data with the signal space transformation robust multi-array average algorithm for background reduction, intensity normalization, probe set summarization and log_2_ transformation. The further analysis of the transcriptome data was restricted to gene-level probe sets with annotated NCBI Gene identifier. We also excluded genes that were not expressed by eliminating probe sets with a log_2_ signal intensity of <4 in all samples from both the B-cell and T-cell datasets.

### 2.8 Gene expression analysis by real-time PCR

Seven genes that are preferentially expressed by CD4^+^ cytotoxic T lymphocytes (CD4-CTLs) (55) were selected to evaluate whether they are differentially expressed in responders vs. non-responders to apheresis. Eligible baseline T-cell samples of MS patients from the validation cohort were used for this analysis. The RNA from the enriched CD4^+^ T cells was isolated as described in the previous section. Afterwards, reverse transcription was performed using the High-Capacity cDNA Reverse Transcription Kit (Applied Biosystems). Relative quantification of target cDNA levels by real-time polymerase chain reaction (PCR) was performed in triplicate for 45 cycles in a 7900HT Fast Real-Time PCR System (Applied Biosystems) using the TaqMan Universal PCR Master Mix (Applied Biosystems) and inventoried TaqMan assays with FAM dye-labeled minor groove binder probes (Thermo Fisher Scientific) (**Supplementary Table 2**). *GAPDH* was measured as reference gene. Raw C_t_ values were computed automatically using the SDS 2.3 and RQ Manager 1.2 software (Applied Biosystems). We then calculated the mean C_t_ value of each triplicate, normalized the data to the expression of the reference gene (ΔC_t_ method) and transformed the data to the linear scale using the equation 2^−ΔCt^×1000.

### 2.9 Statistical methods

The data were analyzed in R version 4.1.2. The sociodemographic, clinical and medication data of the patients were summarized by descriptive statistics. For this purpose, counts and percentages were calculated for categorical variables, and means, standard deviations (SDs), medians and ranges were calculated for numerical variables. This was done using the valid (i.e., non-missing) data of all patients as well as distinct subgroups in each cohort. For comparing groups of patients, e.g., responders and non-responders to apheresis, we used two-sample two-tailed Welch *t*-tests and Mann-Whitney *U* tests for metric data and Fisher exact tests and chi-squared tests for categorical data. The Shapiro-Wilk test was used to determine whether the data was normally distributed. The significance level was set at α=0.05.

The data from the peptide-based ELISAs, flow cytometry, transcriptome profiling and real-time PCR quantification were analyzed basically in the same manner. Means, SDs and standard errors were generally used to describe the data. The baseline and follow-up data were compared using paired *t*-tests, thereby controlling for individual-level variability by focusing on the differences within each subject. The baseline data of responders and non-responders to relapse therapy were compared with Welch *t*-tests, not assuming equal variances. The interaction “response × time” was tested for significance using repeated measures analysis of variance (ANOVA) using the rstatix R package. We further calculated *t*-test effect sizes (Cohen’s *d*) and partial eta-squared (η_p_^2^) estimates for the interaction terms. It should be noted, however, that the data were normally distributed for some but not all peptides, cell types and genes. We have therefore checked the key results with the corresponding non-parametric tests (Wilcoxon signed-rank test and Mann-Whitney *U* tests). Visualizations of the data included bar charts and boxplots.

In the analysis of the transcriptome data, mean differences are presented as log_2_ fold changes (FC), as the data are in log_2_ scale, and the false discovery rate (FDR) approach was applied to adjust the *p-*values for multiple testing (56). The criteria to identify differentially expressed genes (DEGs) were a log_2_FC of >2.0 or <-2.0 and a raw *p*-value of <0.05. The overlap of DEGs was visualized in Venn diagrams. For the genes that were significantly upregulated or downregulated in response to therapeutic apheresis in CD19^+^ B cells or CD4^+^ T cells, we computed their enrichment in Reactome pathways (57). This was done by calculating Fisher’s exact test *p*-values and odds ratios (ORs) and ranking the 10 most significant pathways using the enrichplot R package. Another gene set enrichment analysis was performed with the fgsea R package to test whether MS patients who did not adequately respond to therapeutic apheresis show an elevated expression of the CD4-CTL gene signature (55) in CD4^+^ T cells at baseline as compared to clinical responders.

## 3 Results

### 3.1 Patient characteristics and clinical outcomes

The main study cohort comprised 33 patients with MS having a relapse (**Table 1**). For relapse treatment, the patients received either high-dose methylprednisolone (*n*=27) or therapeutic apheresis (*n*=3 with plasma exchange and *n*=3 with immunoadsorption), the latter usually in the escalation treatment phase. The mean age of the patients was 40.6 ± 12.7 years, and the ratio of women to men was approximately 2:1. The patients’ neurological impairment was at a median EDSS score of 3.0. The DMT of the patients was very heterogeneous (*n*=13 without DMT, *n*=20 with DMT): There was no single DMT used by 4 or more patients (*n*=3 each for dimethyl fumarate, glatiramer acetate and teriflunomide). When comparing patients receiving GCs and patients receiving apheresis, there was no statistically significant difference, except for relapse therapy (phase) according to which the patients were categorized. There were a total of 25 responders (R) and 8 non-responders (NR) to the relapse treatment. Among the R, however, only 5 patients showed an improvement in the EDSS score (*n*=11 unchanged, *n*=9 with missing value). There was no significant difference in the comparisons of R vs. NR to GCs or apheresis. It is nevertheless worth noting that the NR to apheresis (*n*=2) had a relatively long disease duration of 12 or more years, while the R to apheresis (*n*=4) were diagnosed with MS on average 4.5 years ago (*p*=0.240).

**Table 1:** Overview of the main study cohort with subgroup comparisons.

Characteristic	Total	Patients with MS receiving glucocorticoids	Patients with MS receiving apheresis	GCs vs. apheresis *p*-value	GCs R vs. NR *p*-value	Apheresis R vs. NR *p*-value
Age (years), mean ± SD	40.6 ± 12.7	41.0 ± 13.5	38.8 ± 9.1	0.636 ^t^	0.864 ^t^	0.708 ^t^
Sex, *n* (%)				0.640 ^Fi^	1.000 ^Fi^	0.333 ^Fi^
Men	10 (30.3)	9 (33.3)	1 (16.7)			
Women	23 (69.7)	18 (66.7)	5 (83.3)			
Study center, *n* (%)				0.068 ^chi^	0.617 ^chi^	0.223 ^chi^
Berlin	6 (18.2)	6 (22.2)	0 (0.0)			
Greifswald	15 (45.5)	14 (51.9)	1 (16.7)			
Jena	5 (15.2)	3 (11.1)	2 (33.3)			
Rostock	7 (21.2)	4 (14.8)	3 (50.0)			
Current smoker^1^, *n* (%)				1.000 ^Fi^	1.000 ^Fi^	0.467 ^Fi^
No	18 (58.1)	14 (56.0)	4 (66.7)			
Yes	13 (41.9)	11 (44.0)	2 (33.3)			
Body mass index^1^, mean ± SD	24.9 ± 4.8	24.2 ± 4.3	27.7 ± 5.8	0.206 ^t^	0.909 ^t^	0.752 ^t^
Disease course, *n* (%)				1.000 ^Fi^	1.000 ^Fi^	–
CIS/RRMS	30 (90.9)	24 (88.9)	6 (100.0)			
SPMS	3 (9.1)	3 (11.1)	0 (0.0)			
Disease duration (years), median (range)	11 (0-22)	11 (0-22)	7 (0-21)	0.606 ^U^	0.682 ^U^	0.240 ^U^
Comorbidities, *n* (%)				1.000 ^Fi^	0.182 ^Fi^	1.000 ^Fi^
No	14 (42.4)	12 (44.4)	2 (33.3)			
Yes	19 (57.6)	15 (55.6)	4 (66.7)			
Relapses in the past 2 years, median (range)	1 (0-4)	1 (0-4)	1.5 (0-4)	0.402 ^U^	0.598 ^U^	0.812 ^U^
EDSS score at relapse^2^, median (range)	3.0 (1.5-7.5)	3.0 (1.5-7.5)	3.25 (1.5-7.5)	0.696 ^U^	0.951 ^U^	0.133 ^U^
Relapse presentation, *n* (%)				1.000 ^Fi^	0.182 ^Fi^	0.467 ^Fi^
Monosymptomatic	19 (57.6)	15 (55.6)	4 (66.7)			
Polysymptomatic	14 (42.4)	12 (44.4)	2 (33.3)			
Symptomatic phenotype^2^, *n* (%)				0.296 ^Fi^	1.000 ^Fi^	–
New	25 (78.1)	19 (73.1)	6 (100.0)			
Recurring	7 (21.9)	7 (26.9)	0 (0.0)			
Relapse therapy, *n* (%)				**<0.001** ^chi^	–	1.000 ^Fi^
Methylprednisolone	27 (81.8)	27 (100.0)	0 (0.0)			
Immunoadsorption	3 (9.1)	0 (0.0)	3 (50.0)			
Plasma exchange	3 (9.1)	0 (0.0)	3 (50.0)			
Relapse therapy phase^2^, *n* (%)				**<0.001** ^Fi^	–	0.200 ^Fi^
Initiation	28 (87.5)	27 (100.0)	1 (20.0)			
Escalation	4 (12.5)	0 (0.0)	4 (80.0)			
Dose of methylprednisolone (g), median (range)	–	5.0 (3.0-5.0)	–	–	0.329 ^U^	–
Number of apheresis sessions, median (range)	–	–	5 (4-8)	–	–	1.000 ^U^
Clinical outcome, *n* (%)				0.616 ^Fi^	**<0.001** ^Fi^	0.067 ^Fi^
Response	25 (75.8)	21 (77.8)	4 (66.7)			
Non-response	8 (24.2)	6 (22.2)	2 (33.3)			

From a total of 33 patients with relapsing MS, blood samples were obtained before and after relapse treatment with either GCs or therapeutic apheresis. The data were compared according to type of therapy and clinical response. Significant differences (*p*<0.05) are indicated in bold.

^1^two cases with missing data were not considered, ^2^one case with missing data was not considered. ^chi^, chi-squared test; ^Fi^, Fisher’s exact test; ^t^, Welch’s *t*-test; ^U^, Mann-Whitney *U* test.

CIS, clinically isolated syndrome; EDSS, Expanded Disability Status Scale; GC, glucocorticoid; MS, multiple sclerosis; NR, non-responders; R, responders; RRMS, relapsing-remitting multiple sclerosis; SD, standard deviation; SPMS, secondary progressive multiple sclerosis.

The validation cohort included 30 patients (*n*=21 with MS, *n*=9 with NMOSD) who received therapeutic apheresis to manage a relapse. The patients with NMOSD were significantly older and had a significantly higher median EDSS score than the patients with MS (**Table 2**). The NMOSD group also had a higher proportion of women (88.9% vs. 52.4%) and more NR to apheresis (55.6% vs. 23.8%) than the MS group, though not significantly. There were a total of 20 R and 10 NR. However, there was no significant difference in the comparison of R vs. NR among the parameters recorded, including time to apheresis since relapse onset.

**Table 2:** Overview of the validation cohort with subgroup comparisons.

Characteristic	Total	Patients with MS receiving apheresis	Patients with NMOSD receiving apheresis	MS vs. NMOSD *p*-value	MS R vs. NR *p*-value	NMOSD R vs. NR *p*-value
Age (years), mean ± SD	41.8 ± 15.9	35.0 ± 9.0	57.9 ± 17.2	**0.004** ^t^	0.858 ^t^	0.584 ^t^
Sex, *n* (%)				0.100 ^Fi^	0.311 ^Fi^	1.000 ^Fi^
Men	11 (36.7)	10 (47.6)	1 (11.1)			
Women	19 (63.3)	11 (52.4)	8 (88.9)			
Study center, *n* (%)				0.285 ^chi^	0.269 ^chi^	1.000 ^Fi^
Bochum	22 (73.3)	15 (71.4)	7 (77.8)			
Hannover	4 (13.3)	2 (9.5)	2 (22.2)			
Regensburg	4 (13.3)	4 (19.0)	0 (0.0)			
Disease, *n* (%)				**<0.001** ^Fi^	–	–
CIS/RRMS	21 (70.0)	21 (100.0)	0 (0.0)			
NMOSD	9 (30.0)	0 (0.0)	9 (100.0)			
EDSS score at relapse^1^, median (range)	3.5 (1.5-8.5)	3.5 (1.5-6.5)	7.25 (3.0-8.5)	**0.004** ^U^	0.659 ^U^	0.663 ^U^
Time to apheresis (days)^1^, median (range)	22 (11-58)	22 (12-50)	21.5 (11-58)	0.919 ^U^	0.406 ^U^	0.561 ^U^
Relapse therapy, *n* (%)				0.933 ^chi^	0.885 ^chi^	0.155 ^chi^
Immunoadsorption	16 (53.3)	11 (52.4)	5 (55.6)			
Plasma exchange	6 (20.0)	4 (19.0)	2 (22.2)			
Type of apheresis not specified	8 (26.7)	6 (28.6)	2 (22.2)			
Relapse therapy phase^2^, *n* (%)				–	–	–
Escalation	29 (100.0)	20 (100.0)	9 (100.0)			
Clinical outcome, *n* (%)				0.115 ^Fi^	**<0.001** ^Fi^	**0.008** ^Fi^
Response	20 (66.7)	16 (76.2)	4 (44.4)			
Non-response	10 (33.3)	5 (23.8)	5 (55.6)			

From a total of 30 patients with MS or NMOSD, blood samples were obtained before and after relapse treatment with therapeutic apheresis. The data were compared according to disease and clinical response. Significant differences (*p*<0.05) are indicated in bold.

^1^two cases with missing data were not considered, ^2^one case with missing data was not considered. ^chi^, chi-squared test; ^Fi^, Fisher’s exact test; ^t^, Welch’s *t*-test; ^U^, Mann-Whitney *U* test.

CIS, clinically isolated syndrome; EDSS, Expanded Disability Status Scale; MS, multiple sclerosis; NMOSD, neuromyelitis optica spectrum disorder; NR, non-responders; R, responders; RRMS, relapsing-remitting multiple sclerosis; SD, standard deviation.

### 3.2 Changes in antibody responses following relapse treatment

The follow-up blood samples were taken on average 4.5 ± 3.3 days (main study cohort) and 9.0 ± 2.3 days (validation cohort) after baseline. The plasma levels of Ig isotypes remained unchanged after GC therapy (mean decrease <5%), while they fell sharply after apheresis (mean decrease >60%) (**Supplementary Table 3**). In fact, reduced concentrations of IgA, IgG and IgM were seen in all MS patients who underwent apheresis (*n*=6), with the decrease in IgG levels being highly significant (from 4.21 to 0.82 g/L or -81% on average, *p*=0.002).

**Figure 2:**
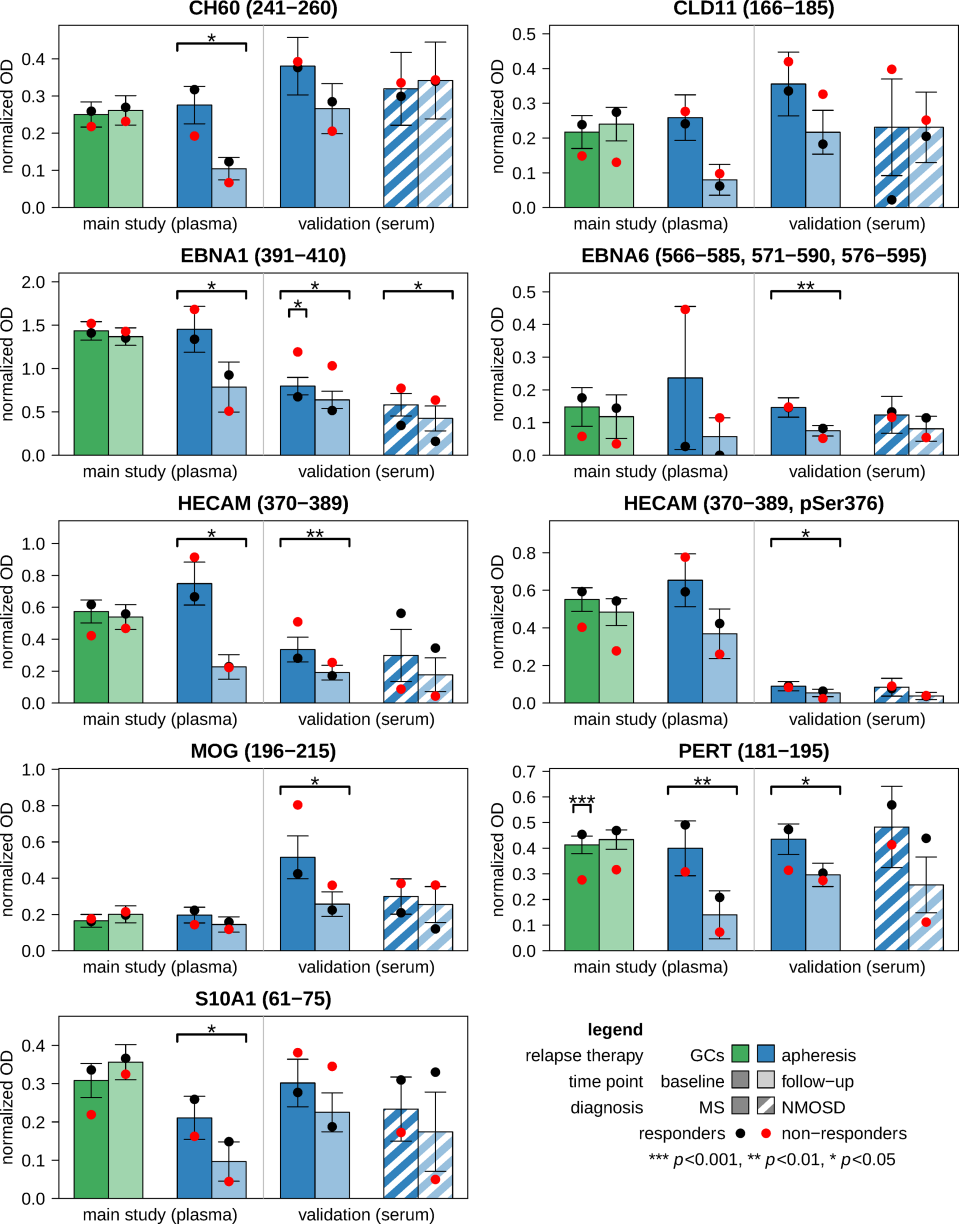
Antibody reactivities against linear epitopes before and after relapse treatment. ELISA measurements were performed using the plasma samples from the main study cohort (*n*=33 patients) and the serum samples from the validation cohort (*n*=30 patients). From each patient, two samples were obtained: one prior to relapse therapy (i.e., baseline) and another one after approximately one week (i.e., follow-up). The bar charts show the mean normalized OD values for those antigen peptides that were analyzed in both plasma and serum. The peptides are specified by the protein identification code according to the UniProt database and the region(s) within the main protein isoform. Error bars indicate standard errors. Black and red dots represent the means of responders and non-responders, respectively. Significance is reported above the brackets. Changes in antibody reactivities were evaluated by paired *t*-tests, and baseline differences between responders and non-responders were evaluated by Welch *t*-tests. The full results are tabulated in **Supplementary Table 3** (main study) and **Supplementary Table 4** (validation). GC, glucocorticoid; ELISA, enzyme-linked immunosorbent assay; MS, multiple sclerosis; NMOSD, neuromyelitis optica spectrum disorder; OD, optical density.

The plasma samples from the main study cohort were used to measure antibody reactivities against 12 peptides from potential protein antigens (**Supplementary Table 1**). In the following, the peptides are consistently named with the UniProt database (58) protein identification code, which has at most 5 alphanumeric characters, and the amino acid positions. The highest OD values were detected for EBNA1 (391-410), followed by CRYAB (3-17) and HECAM (370-389), both of which have overlapping epitopes with the EBNA1 (391-410) sequence (“RRPFF” and “SPPR”, respectively). After GC therapy, the IgG reactivities against all peptides did not change much (|*d*|<0.5 and *p*>0.05, also in non-parametric tests). In contrast, after therapeutic apheresis, a large decrease (*d*<-0.8) in antibody reactivity was observed for 8 peptides and a medium decrease (*d*<-0.5) for 3 peptides (*d*=-0.48 for MOG (196-215)). Despite the small sample size (*n*=6 patients), the significance level was reached for 5 of the 12 peptides (**Figure 2**; **Supplementary Table 3**).

Additional ELISA tests were performed with 9 of the 12 peptides using the sera of the validation cohort. In the subgroup of patients with MS, all IgG reactivities were reduced after apheresis (*n*=6 peptides with *p*<0.05, also in signed-rank tests) (**Figure 2**; **Supplementary Table 4**). The treatment effects were weaker in NMOSD patients, who generally had lower reactivities at baseline than MS patients (although *p*>0.1). However, the reactivities against EBNA1 (391-410) were also significantly reduced at follow-up in the NMOSD group. EBNA1 (391-410) was thus the only peptide in all 3 apheresis groups (MS main study, MS validation and NMOSD validation) showing a significant decrease in IgG reactivities (in both *t*-tests and signed-rank tests).

### 3.3 Comparison of antibody reactivities between responders and non-responders

In the main study, the plasma levels of IgA, IgG and IgM at baseline and their change during therapy in MS patients who received apheresis were not significantly related to overall symptom improvement. The IgG reactivities against the 12 peptides and their dynamics were also not significantly associated with the clinical response to apheresis (**Supplementary Table 3**). Significance was only reached in cases receiving GC therapy for IgG reactivities against PERT (181-195), which were lower in NR than in R at baseline (**Figure 2**). When analyzing the MS patients of the validation cohort, NR showed higher anti-EBNA1 (391-410) reactivities before apheresis than R (*d*=1.38, *p*<0.05 in *t*-test and *U* test) (**Supplementary Table 4**). Higher reactivities against EBNA1 (391-410) in NR vs. R were also noted in the other 2 apheresis groups (MS main study: *d*=0.61, NMOSD validation: *d*=1.37), but these differences were not statistically significant, possibly due to smaller sample sizes (**Figure 2**). Otherwise, no other peptide reached the significance level for R vs. NR at baseline and with regard to the interaction term “response × time”.

### 3.4 Changes in the composition of immune cell subsets following apheresis

**Figure 3:**
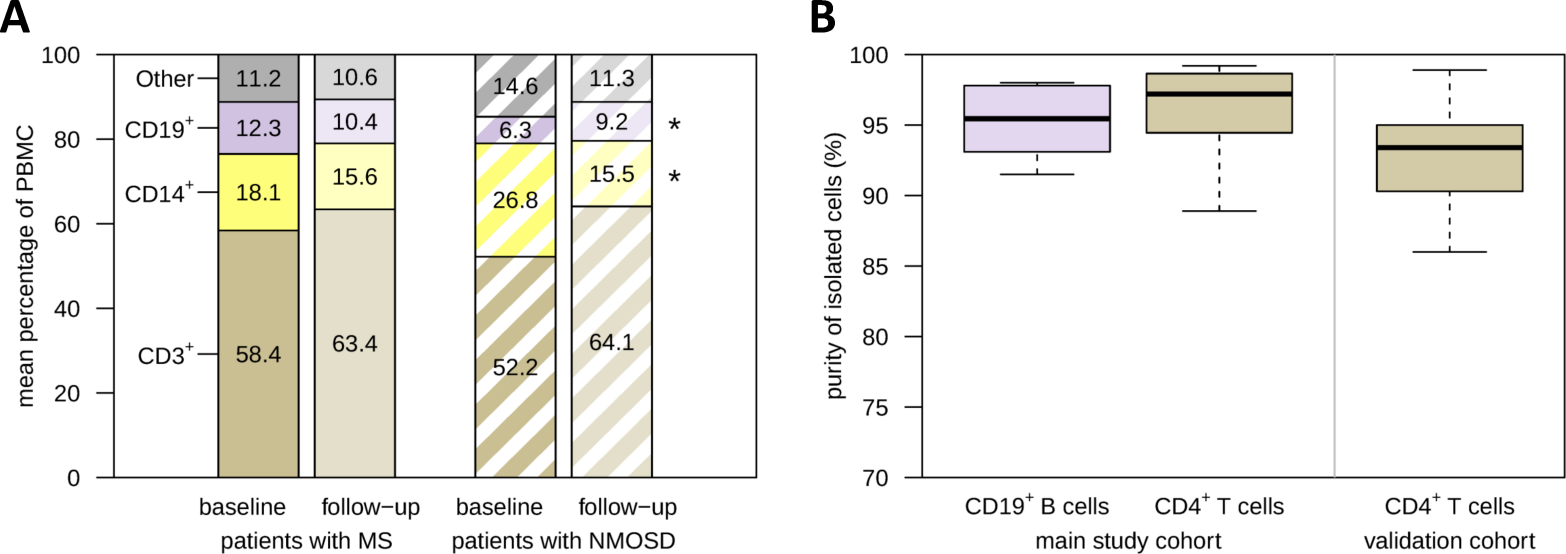
PBMC composition before and after apheresis and enrichment of B cells and T cells. **(A)** Paired PBMC samples from patients with MS (*n*=20) and patients with NMOSD (*n*=9) who received apheresis for the treatment of an acute relapse were analyzed by flow cytometry. The bar chart provides the average relative frequencies of basic immune cell subsets. The cells were gated on live single PBMC. In patients with NMOSD, there was a significant decrease in the proportion of CD14^+^ monocytes and a significant increase in the proportion of CD19^+^ B cells, as revealed by paired *t*-tests. **(B)** CD19^+^ B cells and CD4^+^ T cells were isolated from a subset of MS patient PBMC samples by magnetic separation (main study: *n*=6 B-cell samples and *n*=8 T-cell samples, validation: *n*=19 T-cell samples). Purity was assessed by gating on CD3^-^CD20^+^ and CD3^+^CD4^+^ cells, respectively. RNA from these cells was used for gene expression analyses. *p<0.05. MS, multiple sclerosis; NMOSD, neuromyelitis optica spectrum disorder; PBMC, peripheral blood mononuclear cells.

The proportions of basic PBMC subpopulations were determined for 29 patients (*n*=58 samples) of the validation cohort. The data revealed that therapeutic apheresis was accompanied by moderate shifts in the relative frequencies of immune cell subsets: In NMOSD patients, we observed a significant increase in the percentage of CD19^+^ B cells and a significant decrease in the percentage of CD14^+^ monocytes (**Figure 3A**). Over all patients (*n*=20 with MS and *n*=9 with NMOSD), the decrease in the proportion of monocytes (from 20.8% to 15.6% on average) and the increase in the proportion of CD3^+^ T cells (from 56.5% to 63.6% on average) were significant. However, the percentages of these cell types at baseline and their change from baseline to follow-up were not significantly associated with the clinical response to apheresis therapy (*p*>0.05).

The purity of the isolated CD19^+^ B cells (*n*=6 samples) and CD4^+^ T cells (*n*=8 samples) for the transcriptome profiling (main study) was at least 88.9% (**Figure 3B**). The enrichment of CD4^+^ T cells for the validation by real-time PCR (*n*=19 samples) was lower, but still averaged 92.1%. The B-cell and T-cell samples were therefore considered eligible for the gene expression analyses.

### 3.5 Transcriptome changes in B cells and T cells induced by apheresis

We profiled the transcriptome of CD19^+^ B cells from 3 MS patients (*n*=6 samples) and of CD4^+^ T cells from 4 MS patients (*n*=8 samples) receiving apheresis as relapse therapy. The RNA integrity numbers (59) of the isolated RNA averaged 8.3 ± 0.4 (B-cell samples) and 8.4 ± 0.3 (T-cell samples). The Clariom D arrays passed all quality control measures, including the expected increases in signal values of the labeling controls and hybridization controls. The data processing yielded expression values for a total of 135750 gene-level probe sets (i.e., transcript clusters). We restricted our analysis to 24322 transcripts with NCBI Gene identifier, and we excluded transcripts that were not expressed in the B-cell samples (*n*=6353) or T-cell samples (*n*=4222) (maximum signal <4). The raw and processed data have been deposited in the Gene Expression Omnibus (GEO) database under accession number GSE272973.

**Figure 4:**
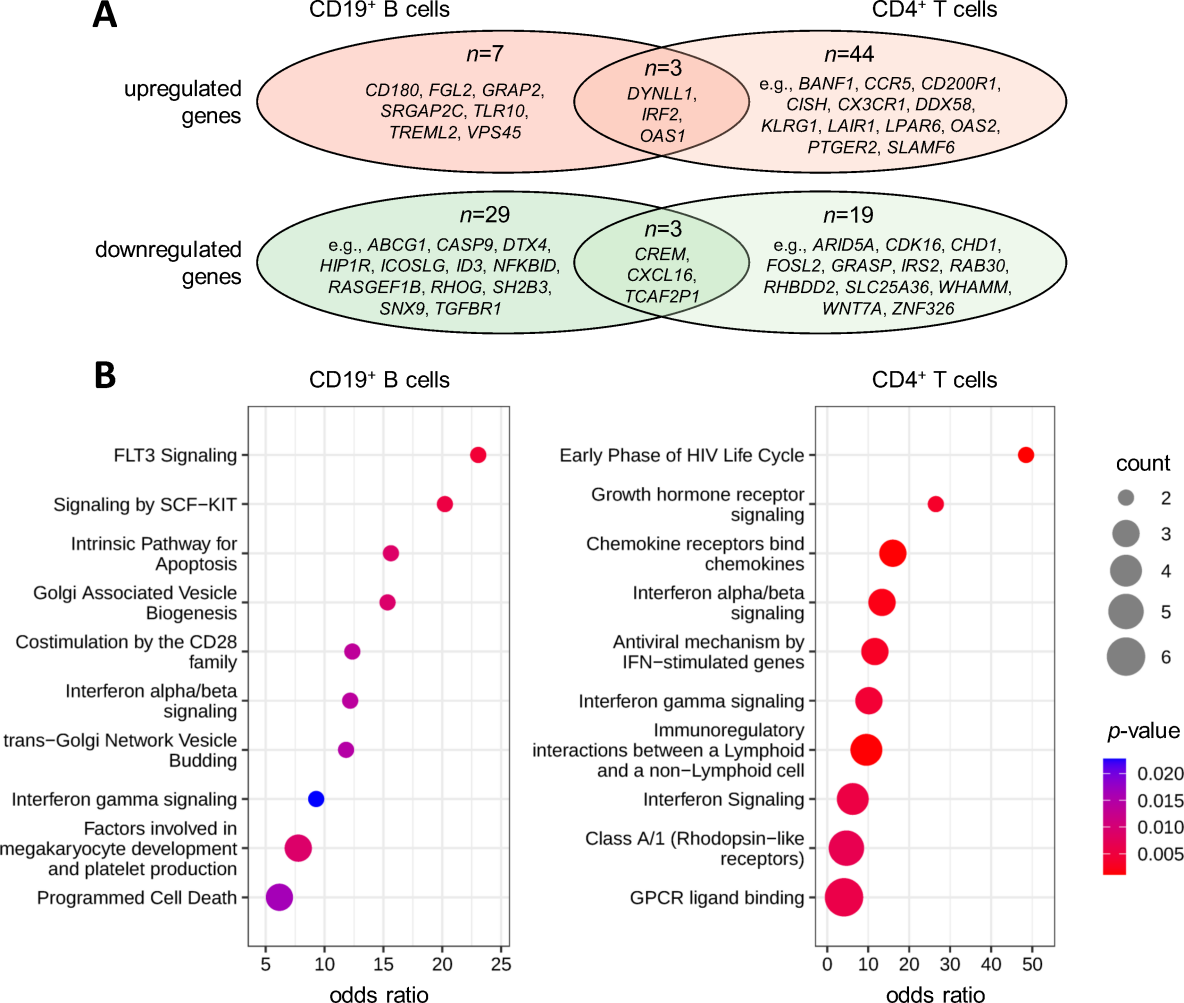
Gene expression changes in response to therapeutic apheresis in patients with MS. Peripheral blood was taken from MS patients in relapse before and after receiving apheresis. The transcriptome profiles of CD19^+^ B cells (*n*=3 patients) and CD4^+^ T cells (*n*=4 patients) were then measured to identify up- or downregulated genes. **(A)** Overlap of DEGs. In total, 42 genes in B cells (**Supplementary Table 5**) and 69 genes in T cells (**Supplementary Table 6**) were filtered with paired *t*-test *p*<0.05 and |log_2_FC|>2.0. There were 3 genes with significantly increased expression and 3 genes with significantly reduced expression in both cell populations. **(B)** Pathway enrichment analysis. The top 10 enriched Reactome pathways are shown for the gene lists obtained for B cells and CD4^+^ T cells. Count specifies the number of DEGs in the pathway. Statistical significance and odds ratios were calculated by Fisher exact tests. DEG, differentially expressed gene; FC, fold change; MS, multiple sclerosis.

By comparing the gene expression at baseline and follow-up, moderate transcriptome changes were detected in response to therapeutic apheresis. For CD19^+^ B cells, 42 DEGs were filtered (10 genes as upregulated and 32 genes as downregulated) (**Supplementary Table 5**). For CD4^+^ T cells, 69 DEGs were filtered (47 genes as upregulated and 22 genes as downregulated) (**Supplementary Table 6**). Six gene transcripts were differentially expressed with |log_2_FC|>2.0 and *p*<0.05 in both CD19^+^ B cells and CD4^+^ T cells (**Figure 4A**). However, no gene reached an FDR-adjusted *p*-value <0.05.

The pathway analysis revealed significant enrichments of the DEGs in interferon (IFN) signaling, with ORs between 6.2 and 13.4 for B cells and CD4^+^ T cells (**Figure 4B**). This was in part attributable to the increased expression of *IRF2* and *OAS1* in both cell types (**Supplementary Tables 5**, **6**). The gene products of the DEGs were also overrepresented in costimulation by the CD28 family (OR=12.4 for B cells) and immunoregulatory interactions between lymphoid and non-lymphoid cells (OR=9.5 for CD4^+^ T cells). Furthermore, genes involved in programmed cell death (e.g., *CASP9* and *DYNLL1*) and hematopoietic cell differentiation (e.g., *GRAP2* and *SH2B3*) were enriched in the DEGs from the B-cell dataset (ORs up to 15.6 and 23.1, respectively). Chemokine receptors (e.g., *CCR5* and *CX3CR1*) and rhodopsin-like receptors (e.g., *LPAR6* and *PTGER2*) were enriched in the DEGs from the T-cell dataset (ORs up to 16.0).

### 3.6 Association of gene expression levels with response to apheresis therapy

**Figure 5:**
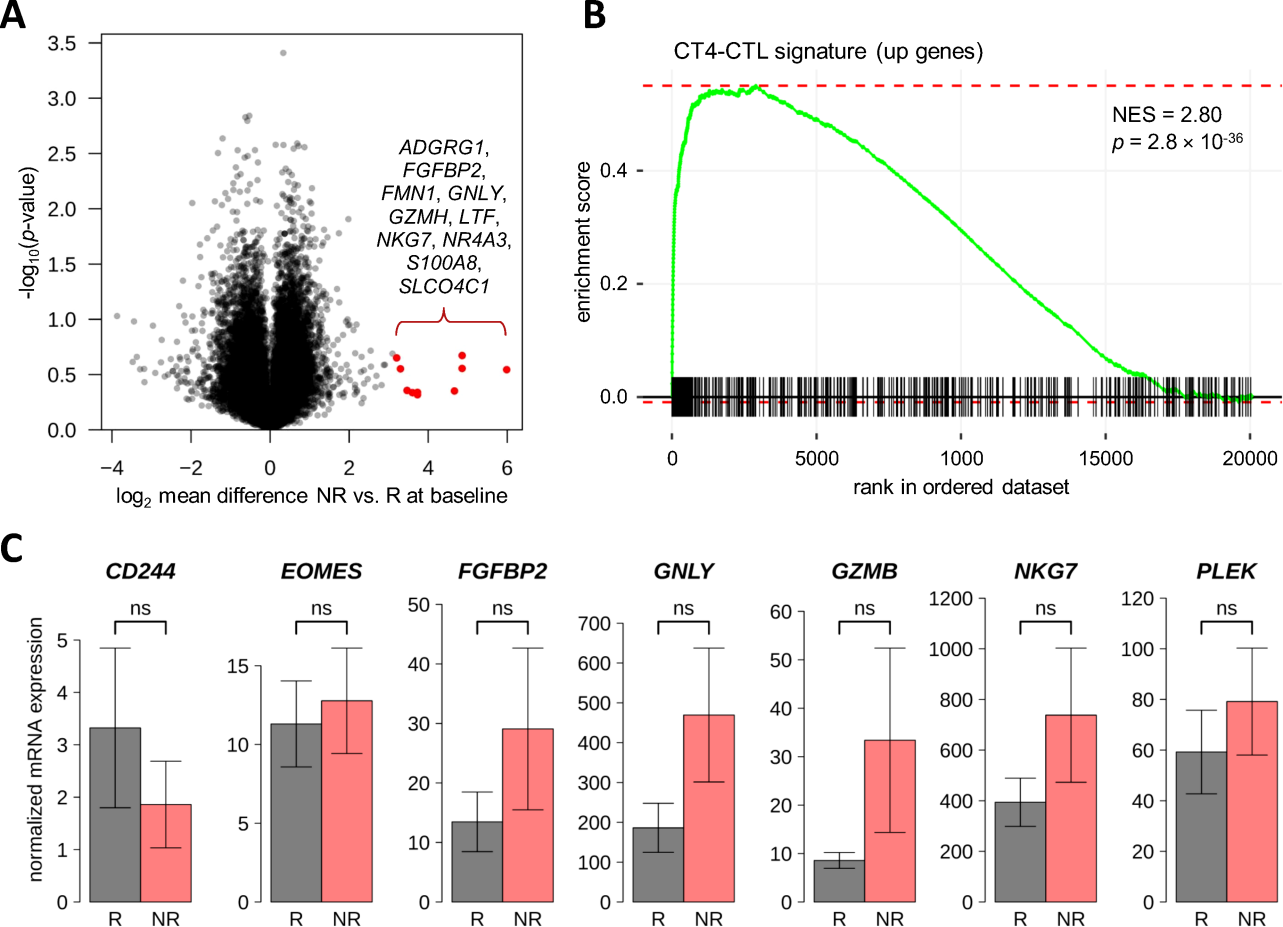
Search for differentially expressed genes between responders and non-responders to therapeutic apheresis in CD4^+^ T cells. **(A)** The transcriptome of CD4^+^ T cells from four MS patients in relapse was measured before and after apheresis. When comparing R (*n*=2) vs. NR (*n*=2) for differential gene expression at baseline or differential change in expression, there was no gene with FDR-adjusted *p*-value <0.05. However, some genes were much higher expressed in NR at baseline. The top 10 autosomal genes are highlighted in the volcano plot. **(B)** The higher expressed genes pointed to an overabundance of CD4-CTLs in NR, which could be verified by a gene set enrichment analysis using the signature from Patil et al. (55). **(C)** A confirmatory analysis by real-time PCR was performed using baseline blood samples from MS patients of the independent validation cohort (*n*=14 R and *n*=5 R). Seven transcripts that showed higher expression in NR vs. R in the transcriptome dataset and that are overexpressed in CD4-CTLs (55) were tested. However, none of these transcripts was significantly differentially expressed in R vs. NR of the validation cohort (Welch *t*-test *p*-values >0.1). The bar charts show the mean normalized mRNA levels (in linear scale) with standard errors. CD4-CTL, CD4^+^ cytotoxic T lymphocyte; FDR, false discovery rate; MS, multiple sclerosis; NES, normalized enrichment score; NR, non-responders; ns, not significant; PCR, polymerase chain reaction; R, responders.

A valid comparison of the transcriptome data according to clinical outcome of apheresis treatment was not feasible due to the limited number of cases and quasi-complete separation by sex (1 NR vs. 2 R for B cells and 2 NR vs. 2 R for T cells, one male patient per dataset). After FDR-based correction for multiple testing, there was no gene with *p*<0.05 for R vs. NR at baseline or for the interaction “response × time”. We nevertheless inspected the T-cell dataset for non-significant expression differences of autosomal genes (*n*=19265) between R and NR. This revealed that some genes were considerably higher expressed in NR than in R before relapse therapy (**Figure 5A**). Among the top 10 of these genes (log_2_ mean difference >3.20, which means >9-fold higher expression in NR *vs*. R) were 5 genes that have been described as CD4-CTL-related genes (55): *FGFBP2*, *GNLY*, *GZMH*, *NKG7* and *SLCO4C1* (log_2_ mean difference >2.18 for CD4-CTLs vs. central memory T cells). A gene set enrichment analysis confirmed that genes that were found to be more abundantly expressed in CD4-CTLs (*n*=517) (55) were typically expressed at higher levels in NR than in R at baseline (*p*=2.8×10^-36^ and normalized enrichment score =2.80) (**Figure 5B**).

For the verification of a potential gene expression signature that may predict clinical response to therapeutic apheresis, 7 genes were selected. These genes were more strongly expressed in the T-cell transcriptome data in NR vs. R prior to apheresis, and an elevated expression of these genes is characteristic for CD4-CTLs (55). The real-time PCR analysis was conducted with baseline T-cell samples from 19 MS patients of the validation cohort who underwent apheresis to manage an acute relapse. In the comparison of R (*n*=14) and NR (*n*=5), none of the 7 genes (*CD244*, *EOMES*, *FGFBP2*, *GNLY*, *GZMB*, *NKG7* and *PLEK*) reached the significance level of α=0.05 in parametric or non-parametric tests. However, for 6 of the 7 CD4-CTL-related genes (all but *CD244*), the mean mRNA level was at least nominally higher in NR than in R in the real-time PCR data (**Figure 5C**). Particularly high expression levels were measured for *NKG7*.

## 4 Discussion

Acute relapses in MS and NMOSD are strongly associated with permanent disability accrual (40, 60). This has two major implications: First, an effective relapse prevention should be ensured via DMTs or long-term immunotherapies (25, 26). Second, a prompt and adequate management of (breakthrough) relapses is indicated to enhance remission and maintain patients’ quality of life. The use of therapeutic apheresis for treating exacerbations of MS dates back to first attempts in the 1980s (61, 62), and it is still a recommended option for patients who do not respond to or do not tolerate high-dose GCs (30, 32). However, we still have only an incomplete understanding of the range of immune parameters that are modulated by this procedure, and there is a lack of prognostic biomarkers that could guide treatment decisions. The present study focused on exploring how (auto)antibody reactivities and gene expression levels are altered following apheresis and whether they are associated with clinical outcomes.

Our analyses were based on two independent patient cohorts. The patients showed similar characteristics (e.g., in terms of age and sex distribution, disease duration and degree of disability) as patients with relapsing MS or NMOSD in large German registries (63–65). Compared to the patients with MS, the patients with NMOSD exhibited a higher proportion of women and on average a higher age as well as a higher EDSS score, which is in line with the literature (66). However, only 4 of the 9 NMOSD patients (44.4%) were classified as responders, whereas the response rate to apheresis has been described to be ~70-100% (34, 38). It should be noted that the criteria for assessing clinical response to therapy differ in other studies (43-47) and also for the two cohorts of the present study. Moreover, it is unknown to which degree spontaneous recovery or a delayed effect of an initial GC treatment may contribute to clinical improvement following apheresis. In the analysis of the sociodemographic and clinical data, we could not detect any significant difference in the comparisons of R vs. NR to GCs or apheresis. Among patients with MS who received apheresis, disease duration was somewhat higher in NR than in R, which corresponds to a previous study that demonstrated an inverse correlation between disease duration and improvement in the EDSS score after therapeutic plasma exchange (36). It has also been reported that a longer time to apheresis is significantly associated with an incomplete remission (36, 38, 67). For the patients in our main study cohort, the number of days since relapse onset was unfortunately not documented, and in the validation cohort, the delay to apheresis was not significantly different between R and NR.

The plasma concentrations of IgA, IgG and IgM and the IgG reactivities against the 12 peptides were not much affected by the relapse therapy with methylprednisolone (*p*>0.05). In contrast, MS patients who received therapeutic apheresis showed a clear decrease in the Ig levels (>60%) and in all measured antibody reactivities in serum and plasma (*d*<-0.2). For 3 peptides, the significance level was reached in both the main study cohort and the validation cohort: EBNA1 (391-410), HECAM (370-389) (also known as GlialCAM) and PERT (181-195) (also known as TPO). The antibody reactivities against these 3 peptides were also substantially reduced in patients with NMOSD after apheresis (*d*<-0.5), with EBNA1 (391-410) being the only one with *p*<0.05. Anti-EBNA1 IgG seropositivity and especially IgG responses to the immunodominant region 385-420 of EBNA1 are well-known to be strongly associated with MS risk (53, 68–71). For the other selected peptides, increased antibody reactivities have been previously demonstrated in serum, plasma or cerebrospinal fluid of individuals with MS compared to controls (12-14, 22, 52). However, our study is the first in which the antibody repertoire against epitopes of EBV proteins and suspected autoantigens has been investigated in the course of a short-term relapse therapy. Of note, the immune reaction against the HECAM peptide is believed to result from cross-reactive anti-EBNA1 antibodies due to the shared amino acid sequence “SPPR”, but our data did not confirm the finding by Lanz et al. according to which phosphorylation at Ser376 may increase antibody binding affinity (13).

EBNA1 (391-410) was the only peptide for which a differential antibody response was observed in R vs. NR to apheresis: The baseline anti-EBNA1 (391-410) IgG reactivities were on average higher in the non-responders of all 3 apheresis groups (*d*>0.6 in MS main study, MS validation and NMOSD validation), though significance was only reached in the analysis of MS patients of the validation cohort. This suggests that an elevated immune response against this EBNA1 fragment is associated with worse disease outcomes, which may guide future decisions regarding apheresis treatment. Earlier studies already reported that the anti-EBNA1 IgG titer is a significant predictor of T2 lesion volume change, disability progression and conversion from CIS to clinically definite MS (72, 73). However, a review on the clinical utility of EBV-directed antibodies in MS concluded that anti-EBNA1 Ig levels may reflect recent inflammatory disease activity and that they decrease following the initiation of certain DMTs, but that their use as prognostic biomarkers in clinical practice currently remains limited due to the lack of methodological precision, reliability and validation (69). Therefore, it is important to further develop and standardize appropriate assays and to further elucidate how anti-EBNA1 antibody levels in combination with other environmental, lifestyle and genetic factors are linked to disease pathophysiology.

We found that apheresis was associated with changes in the relative frequencies of CD14^+^ monocytes, CD19^+^ B cells and CD3^+^ T cells in PBMC, which, however, were not related to the clinical response of the patients. In a study by Pfeuffer et al., the shifts in immune cell subsets during the treatment of MS relapses have been explored in greater detail (45). Their data indicated that immunoadsorption causes a non-significant increase in the numbers of CD4^+^ T cells and CD8^+^ T cells and a profound reduction in the activation marker CD69 on both T-cell subsets. The treatment was also shown to result in decreased B-cell counts (e.g., naïve B cells, transitional B cells and memory B cells) compared to baseline. The reduction in B-cell subsets correlated with the improvement in clinical function, at least in those patients who received apheresis already after the first course of GCs (45). Another study postulated that the T helper 1-cell–CD11c^+^ B-cell axis may be associated with the responsiveness to apheresis (46). B cells, particularly memory B cells, are a major target for DMTs in MS (74, 75), and B-cell-depleting therapies have also been shown to be effective in patients with NMOSD (26, 76). Memory B cells form the reservoir for latent EBV infection, which might give rise to cross-reactive immune responses (77, 78). However, the links between circulating B-cell subsets, pathogenic antibodies and therapeutic outcomes, which may differ between MS and NMOSD, remain incompletely understood (76). The clinical value of phenotyping B cells and other populations within PBMC for a more personalized therapy of relapses needs to be evaluated through further research.

The transcriptome analysis revealed that apheresis is associated with gene expression changes in patients with MS, although these results should be interpreted with caution due to the small sample size and the issue of multiple testing. The average transcript level of the identified DEGs was increased by >300% (log_2_FC>2.0) or decreased by >75% (log_2_FC<-2.0) at the end of the therapy. Three genes were upregulated (*DYNLL1*, *IRF2* and *OAS1*) and 3 genes were downregulated (*CREM*, *CXCL16* and *TCAF2P1*) in both CD19^+^ B cells and CD4^+^ T cells. DYNLL1 is a negative regulator of BIM-mediated apoptosis (79). IRF2 is a transcriptional repressor of IFNs and IFN-inducible genes (80), and lower *IRF2* mRNA levels were reported in PBMC from patients with active MS compared to healthy controls (81). OAS1 is a classic biomarker of IFN-β activity (82, 83) and plays a role in the innate cellular antiviral response (84). CREM is a transcriptional regulator of different cytokines (85), and CXCL16 is a chemokine that regulates the migration of CXCR6-expressing leukocytes (86), which may contribute to inflammatory conditions with CNS involvement (87). Several of the other DEGs have similar functions. Accordingly, the pathway analysis revealed significant enrichments of the DEGs in programmed cell death, IFN signaling and the binding of chemokines to chemokine receptors. There is no other dataset in the literature on the transcriptomic effects of therapeutic apheresis. However, a PCR-based study showed that apheresis leads to a reduced expression of *IFNG* and *STAT1* in T helper 1 cells (46). Another study, in which 45 proteins were measured in serum, found that immunoadsorption skews the blood cytokine network and reduces the levels of cytokines necessary for B-cell maturation (45). It yet remains unclear to what extent the gene expression dynamics are attributable to a direct modulation of circulating immune cells or to the removal of soluble factors that regulate cellular activities.

We also examined the T-cell transcriptome data for associations with the patients’ clinical response, again calling for cautious interpretation. A CD4-CTL gene signature was found to be enriched among the genes that were expressed at higher levels in NR than in R prior to apheresis (55). The real-time PCR analysis with CD4^+^ T cells from independent cases then revealed nominally higher baseline mRNA levels in NR vs. R for 6 out of 7 cytotoxicity-related genes. However, these differences were not statistically significant, which might be explained by the fact that multiple factors led to considerable heterogeneity in the data. For instance, the MS patients were treated at different clinical centers with different apheresis techniques to ameliorate different relapse symptoms. Nevertheless, our data point to a possible association of CD4-CTLs with poor relapse recovery. CD4-CTLs, an effector subset of circulating CD4^+^ T cells, are more abundant in the aged immune system (88, 89). They are generated in response to acute and chronic infections with viruses such as EBV, and they are able to kill infected cells in an MHC class II-restricted antigen-specific manner by producing cytolytic molecules and pro-inflammatory cytokines (88, 90). Accumulating evidence implicates CD4-CTLs in the pathomechanisms of MS (91, 92). Especially older MS patients with progressive disease harbor abnormally increased frequencies of CD4-CTLs in the circulation (89, 93). Importantly, the frequency of these cells has been shown to correlate with disease severity and worse clinical outcome (93, 94). By identifying patients who are less likely to respond to relapse therapy, clinicians could tailor treatment plans more effectively. Therefore, the hypothesis that CD4-CTLs may be of prognostic value in the context of therapeutic apheresis is worth investigating further.

The present study has several limitations. For instance, the number of patients was small in some subgroups (e.g., those with NMOSD), and therefore statistical significance could sometimes not be reached despite large effect sizes. Further international efforts are needed to strengthen the generalizability of our results. Another limitation is that the clinical response evaluation differed between the main study cohort and the validation cohort, and we did not inspect other therapeutic outcomes, e.g., imaging findings, adverse effects or benefits from the patients’ perspective (95). Moreover, the blood samples were taken at only two time points, so that it remains unclear how long the observed treatment effects may persist. However, additional follow-up time points would require extra patient visits, which may be difficult to realize in clinical practice. In the analysis of IgG reactivities, only a limited number of candidate (auto)antigens was analyzed, and we tested only for linear epitopes but not conformational epitopes. We also did not measure other soluble factors such as complement constituents that are known to be altered by apheresis (96). The transcriptome profiling was limited to CD19^+^ B cells and CD4^+^ T cells. Therefore, it would be also interesting to study gene expression changes in other cell populations or at the single-cell level. Furthermore, alterations at the transcript level may not correlate with differences in protein expression, and the results cannot be generalized to other disease conditions, such as MOG antibody-associated disease (MOGAD) (97). On the other hand, strengths of the study include the collection of paired blood samples at multiple clinical centers and the use of different analytical methods. Subsequent research may shed further light on whether EBV-specific antibody responses and CD4-CTLs are related to poor relapse recovery despite therapy.

## 5 Conclusion

Our study provides new insights into the effects of therapeutic apheresis in patients with neuroimmunological diseases, particularly MS and NMOSD. Apheresis was associated with markedly reduced (auto)antibody reactivities and a differential expression of genes related to IFN signaling in both B cells and CD4^+^ T cells. These findings underscore the use of apheresis as a valuable intervention to modulate immune responses in these conditions. While no clear and consistent differences emerged between responders and non-responders, our data revealed higher IgG reactivities against EBNA1 (391-410) and a more abundant expression of CD4-CTL-related genes in CD4^+^ T cells prior to apheresis in patients who did not exhibit clinical improvement. These factors could serve as potential biomarkers for predicting treatment outcomes in individual patients, enabling more personalized approaches to relapse management. Future research should focus on validating these biomarkers and expanding the scope by exploring the effects of relapse therapy on other (auto)antibody specificities and other immune cell subsets. Such investigations could refine our understanding of the immunological mechanisms underlying MS and NMOSD and further enhance the precision of therapeutic strategies, ultimately improving patient outcomes.

## Data Availability

The datasets presented in this study can be found in online repositories. The names of the repository/repositories and accession number(s) can be found below: https://www.ncbi.nlm.nih.gov/geo/, GSE272973.
