# Supplementary material for: Apheresis for the treatment of relapses in MS and NMOSD: reduced antibody reactivities, gene expression changes and potential clinical response indicators
Source: Front Immunol. 2025 Jan 30;16:1531447. doi: 10.3389/fimmu.2025.1531447 (PMC11821495; doi:10.3389/fimmu.2025.1531447)
Supplement: Supplementary file 1 [file DataSheet1.pdf]

Supplementary Information for

Apheresis for the treatment of relapses in MS and NMOSD: Effects on antibody reactivities and gene expression and potential clinical response indicators

Michael Hecker[1], Brit Fitzner[1,†], Isis Ludwig-Portugall[2,†], Friederike Bohne[2], Edmar Heyland[2], Juliane Klehmet[3], Matthias Grothe[4], Matthias Schwab[5], Alexander Winkelmann[1], Stefanie Meister[1], Ales Dudesek[1], Hannah Wurm[6], Ilya Ayzenberg[6], Ingo Kleiter[6,7], Corinna Trebst[8], Martin W. Hümmert[8], Bernhard Neumann[9,10], Klaus Eulitz[2], Dirk Koczan[11], Uwe K. Zettl[1]

[1] Division of Neuroimmunology, Department of Neurology, Rostock University Medical Center, Rostock, Germany

[2] Miltenyi Biotec B.V. & Co. KG, Teterow, Germany

[3] Center for Multiple Sclerosis, Department of Neurology, Jüdisches Krankenhaus Berlin, Berlin, Germany

[4] Department of Neurology, University Medicine Greifswald, Greifswald, Germany

[5] Department of Neurology, Jena University Hospital, Jena, Germany

[6] Department of Neurology, St. Josef-Hospital, Ruhr University Bochum, Bochum, Germany

[7] Marianne-Strauß-Klinik, Behandlungszentrum Kempfenhausen für Multiple Sklerose Kranke gGmbH, Berg, Germany

[8] Department of Neurology, Hannover Medical School, Hannover, Germany

[9] Department of Neurology, University of Regensburg, Bezirksklinikum, Regensburg, Germany

[10] Department of Neurology, Donau-Isar-Klinikum Deggendorf, Deggendorf, Germany

[11] Institute of Immunology, Rostock University Medical Center, Rostock, Germany

[†] These authors contributed equally to this work

Supplemental Table 1 : Peptides that were used for measuring antibody reactivities in plasma or serum with ELISAs.

Peptide name	UniProt entry	Gene symbol	Organism	Peptide sequence
CH60 (241-260)	P10809	*HSPD1*	Homo sapiens	DAYVLLSEKKISSIQSIVPA
CLD11 (166-185)	O75508	*CLDN11*	Homo sapiens	VLCLVGGCVILCCAGDAQAF
CRYAB (3-17)	P02511	*CRYAB*	Homo sapiens	IAIHHPWIRRPFFPF
EBNA1 (391-410)	P03211	*EBNA1*	Epstein-Barr virus	SGSPPRRPPPGRRPFFHPVG
EBNA6 (566-585, 571-590, 576-595)	P03204	*EBNA6*	Epstein-Barr virus	PPAAGPPAAGPPAAGPPAAG
HECAM (370-389)	Q14CZ8	*HEPACAM*	Homo sapiens	ATGRTHSSPPRAPSSPGRSR
HECAM (370-389, pSer376)	Q14CZ8	*HEPACAM*	Homo sapiens	ATGRTHpSSPPRAPSSPGRSR
MBP (257-276)	P02686	*MBP*	Homo sapiens	PGFGYGGRASDYKSAHKGFK
MOG (196-215)	Q16653	*MOG*	Homo sapiens	TFDPHFLRVPCWKITLFVIV
PERT (181-195)	P07202	*TPO*	Homo sapiens	YEDGFSQPRGWNPGF
PGAM1 (43-57)	P18669	*PGAM1*	Homo sapiens	QALRDAGYEFDICFT
S10A1 (61-75)	P23297	*S100A1*	Homo sapiens	ELDENGDGEVDFQEY

Twelve peptide sequences were derived from proteins that were previously described as (auto)antigens in the literature. The name of each peptide is composed of the UniProt database entry name of the protein (mnemonic code "X") and the region(s) within the main protein isoform. In case of peptides from Epstein-Barr virus, the sequences were mapped to proteins of strain B95-8.

ELISA = enzyme-linked immunosorbent assay

Supplemental Table 2 : Real-time PCR assays for validating the cytotoxicity-related gene expression signature in CD4^{+} T cells.

Gene symbol	NCBI Gene ID	TaqMan assay	Amplicon length
CD244	51744	Hs00175569_m1	128
EOMES	8320	Hs00172872_m1	81
FGFBP2	83888	Hs00230605_m1	87
GAPDH	2597	Hs99999905_m1	122
GNLY	10578	Hs00246266_m1	80
GZMB	3002	Hs00188051_m1	114
NKG7	4818	Hs01120688_g1	65
PLEK	5341	Hs00950975_m1	89

The transcriptome data (*n*=4 patients with MS) suggested an elevated expression of genes, which are preferentially expressed by cytotoxic CD4^{+} T cells (CD4-CTLs), in non-responders to apheresis as compared to responders. Seven genes and *GAPDH* as reference gene were chosen to validate the potential predictive value of this signature in CD4^{+} T-cell samples from an independent cohort of 19 MS patients in relapse before apheresis treatment.

ID = identifier, NCBI = National Center for Biotechnology Information

Supplemental Table 3 : Dynamics in Ig concentrations and IgG reactivities following MS relapse treatment (main study cohort).

		Mean ± SD		Baseline vs. follow-up		NR vs. R at baseline		Interaction	
**Assay**	***n***	**Baseline**	**Follow-up**	***p*-value**	**Cohen's *d***	***p*-value**	**Cohen's *d***	***p*-value**	**η_p^2**
**Patients with MS receiving glucocorticoids**									
IgA (g/L)	27	1.28 ± 0.62	1.25 ± 0.58	0.682	-0.08	0.537	0.31	**0.019**	0.20
IgG (g/L)	27	6.51 ± 2.09	6.42 ± 2.07	0.800	-0.05	0.471	0.39	**0.034**	0.17
IgM (g/L)	27	0.45 ± 0.22	0.43 ± 0.20	0.590	-0.11	0.850	-0.09	0.785	0.00
CH60 (241-260)	23	0.25 ± 0.16	0.26 ± 0.19	0.665	0.09	0.597	-0.26	0.957	0.00
CLD11 (166-185)	21	0.22 ± 0.22	0.24 ± 0.22	0.396	0.19	0.326	-0.46	0.395	0.04
CRYAB (3-17)	27	0.92 ± 0.74	0.94 ± 0.79	0.423	0.16	0.879	-0.07	0.885	0.00
EBNA1 (391-410)	27	1.43 ± 0.55	1.37 ± 0.52	0.071	-0.36	0.608	0.22	0.713	0.01
EBNA6 (566-585, 571-590, 576-595)	21	0.15 ± 0.27	0.12 ± 0.31	0.624	-0.11	0.189	-0.53	0.952	0.00
HECAM (370-389)	27	0.57 ± 0.37	0.54 ± 0.40	0.287	-0.21	0.140	-0.60	0.186	0.07
HECAM (370-389, pSer376)	27	0.55 ± 0.33	0.48 ± 0.37	0.062	-0.38	0.072	-0.70	0.367	0.03
MBP (257-276)	21	0.13 ± 0.12	0.10 ± 0.12	0.169	-0.31	0.847	-0.10	0.729	0.01
MOG (196-215)	22	0.16 ± 0.17	0.20 ± 0.22	0.163	0.31	0.916	0.06	0.960	0.00
PERT (181-195)	26	0.41 ± 0.17	0.43 ± 0.19	0.402	0.17	**0.001**	-1.34	0.683	0.01
PGAM1 (43-57)	21	0.20 ± 0.27	0.19 ± 0.25	0.886	-0.03	0.732	-0.18	0.786	0.00
S10A1 (61-75)	21	0.31 ± 0.20	0.36 ± 0.21	0.203	0.29	0.199	-0.63	0.395	0.04
**Patients with MS receiving apheresis**									
IgA (g/L)	6	0.89 ± 0.52	0.31 ± 0.21	0.062	-0.98	0.212	-1.10	0.722	0.04
IgG (g/L)	6	4.21 ± 0.41	0.82 ± 1.37	**0.002**	-2.30	0.473	0.58	0.430	0.16
IgM (g/L)	6	0.46 ± 0.24	0.17 ± 0.10	0.063	-0.97	0.498	-0.54	0.649	0.06
CH60 (241-260)	6	0.28 ± 0.12	0.10 ± 0.07	**0.019**	-1.39	0.383	-1.02	0.582	0.08
CLD11 (166-185)	4	0.26 ± 0.13	0.08 ± 0.09	0.124	-1.06	0.844	0.22	1.000	0.00
CRYAB (3-17)	6	0.65 ± 0.66	0.48 ± 0.67	0.106	-0.81	0.499	-0.55	0.122	0.49
EBNA1 (391-410)	6	1.45 ± 0.65	0.79 ± 0.71	**0.024**	-1.30	0.455	0.61	0.075	0.59
EBNA6 (566-585, 571-590, 576-595)	4	0.24 ± 0.44	0.06 ± 0.11	0.348	-0.56	0.520	0.94	0.455	0.30
HECAM (370-389)	6	0.75 ± 0.33	0.23 ± 0.19	**0.011**	-1.60	0.311	0.87	0.427	0.16
HECAM (370-389, pSer376)	6	0.65 ± 0.34	0.37 ± 0.32	0.112	-0.79	0.464	0.60	0.317	0.25
MBP (257-276)	4	0.21 ± 0.18	0.09 ± 0.10	0.152	-0.96	0.130	-4.80	0.166	0.70
MOG (196-215)	6	0.20 ± 0.11	0.14 ± 0.10	0.288	-0.48	0.303	-0.87	0.722	0.04
PERT (181-195)	4	0.40 ± 0.21	0.14 ± 0.19	**0.002**	-5.48	0.561	-0.81	0.415	0.34
PGAM1 (43-57)	4	0.12 ± 0.13	0.02 ± 0.02	0.234	-0.74	0.173	-3.10	0.150	0.72
S10A1 (61-75)	4	0.21 ± 0.11	0.10 ± 0.10	**0.026**	-2.06	0.506	-0.81	0.922	0.01

Blood plasma samples were collected before and after relapse treatment with high-dose glucocorticoids or therapeutic apheresis. The number of patients with paired samples that were considered for the measurements is given in the second column. The average levels and ODs at baseline and follow-up were compared using paired *t*-tests. The baseline data of responders and non-responders were compared with Welch *t*-tests. We also analyzed whether the interaction "response × time" is significant using repeated measures ANOVA. Cohen's *d* and partial eta-squared estimates as measures of effect size are reported in the table. *P*-values <0.05 are indicated in bold.

ANOVA = analysis of variance, Ig = immunoglobulin, MS = multiple sclerosis, NR = non-responders, OD = optical density, R = responders, SD = standard deviation

Supplemental Table 4 : Dynamics in antibody reactivities following apheresis for relapse treatment (validation cohort).

Assay	*n*	Mean ± SD Baseline	Mean ± SD Follow-up	Baseline vs. follow-up *p*-value	Baseline vs. follow-up Cohen's *d*	NR vs. R at baseline *p*-value	NR vs. R at baseline Cohen's *d*	Interaction *p*-value	Interaction η_p^2
**Patients with MS receiving apheresis**									
CH60 (241-260)	21	0.38 ± 0.35	0.27 ± 0.31	0.270	-0.25	0.924	0.05	0.699	0.01
CLD11 (166-185)	21	0.36 ± 0.42	0.22 ± 0.29	0.129	-0.35	0.688	0.20	0.781	0.00
EBNA1 (391-410)	21	0.80 ± 0.46	0.64 ± 0.46	**0.030**	-0.51	**0.013**	1.38	0.995	0.00
EBNA6 (566-585, 571-590, 576-595)	21	0.15 ± 0.14	0.07 ± 0.07	**0.002**	-0.77	0.982	0.01	0.512	0.02
HECAM (370-389)	21	0.34 ± 0.36	0.19 ± 0.21	**0.003**	-0.74	0.208	0.68	0.150	0.11
HECAM (370-389, pSer376)	21	0.09 ± 0.11	0.05 ± 0.09	**0.025**	-0.53	0.898	-0.07	0.366	0.04
MOG (196-215)	21	0.51 ± 0.54	0.26 ± 0.31	**0.025**	-0.53	0.264	0.67	0.343	0.05
PERT (181-195)	21	0.44 ± 0.27	0.30 ± 0.21	**0.028**	-0.52	0.107	-0.69	0.355	0.05
S10A1 (61-75)	21	0.30 ± 0.28	0.23 ± 0.23	0.127	-0.35	0.470	0.38	0.647	0.01
**Patients with NMOSD receiving apheresis**									
CH60 (241-260)	9	0.32 ± 0.29	0.34 ± 0.31	0.881	0.05	0.876	0.11	0.920	0.00
CLD11 (166-185)	9	0.23 ± 0.42	0.23 ± 0.30	1.000	0.00	0.181	1.02	0.189	0.23
EBNA1 (391-410)	9	0.58 ± 0.39	0.43 ± 0.43	**0.011**	-1.09	0.096	1.37	0.661	0.03
EBNA6 (566-585, 571-590, 576-595)	9	0.12 ± 0.17	0.08 ± 0.11	0.210	-0.45	0.892	-0.09	0.525	0.06
HECAM (370-389)	9	0.30 ± 0.49	0.18 ± 0.32	0.127	-0.57	0.242	-0.99	0.247	0.19
HECAM (370-389, pSer376)	9	0.08 ± 0.14	0.04 ± 0.06	0.285	-0.38	0.891	0.09	0.914	0.00
MOG (196-215)	9	0.30 ± 0.29	0.25 ± 0.30	0.630	-0.17	0.415	0.57	0.684	0.03
PERT (181-195)	9	0.48 ± 0.47	0.26 ± 0.33	0.112	-0.60	0.650	-0.31	0.536	0.06
S10A1 (61-75)	9	0.23 ± 0.25	0.17 ± 0.31	0.257	-0.41	0.522	-0.50	0.152	0.27

Serum samples were collected before and after relapse treatment with apheresis from patients with MS or NMOSD. The number of patients with paired samples that were considered for the measurements is given in the second column. The average ODs at baseline and follow-up were compared using paired *t*-tests. The baseline data of responders and non-responders were compared with Welch *t*-tests. We also analyzed whether the interaction "response × time" is significant using repeated measures ANOVA. Cohen's *d* and partial eta-squared estimates as measures of effect size are reported in the table. *P*-values <0.05 are indicated in bold.

ANOVA = analysis of variance, MS = multiple sclerosis, NMOSD = neuromyelitis optica spectrum disorder, NR = non-responders, OD = optical density, R = responders, SD = standard deviation

Supplemental Table 5 : Gene expression changes in peripheral blood CD19$^+$ B cells in response to therapeutic apheresis.

Transcript cluster	Gene symbol	NCBI Gene ID	Mean t0	Mean t1	log_2FC	*p*-value	FDR	R-HSA-9607240	R-HSA-1433557	R-HSA-109606	R-HSA-432722	R-HSA-388841	R-HSA-909733	R-HSA-199992	R-HSA-877300	R-HSA-983231	R-HSA-5357801
TC2100007241.hg.1	*ABCG1*	9619	10.82	8.01	-2.82	**0.029**	0.720										
TC0100013000.hg.1	*CASP9*	842	8.32	6.30	-2.02	**0.046**	0.727			X							X
TC0500013316.hg.1	*CD180*	4064	14.93	17.83	2.90	**0.009**	0.708										
TC0300010643.hg.1	*CMTM6*	54918	12.57	10.29	-2.28	**0.047**	0.727										
TC1000007272.hg.1	*CREM*	1390	11.52	7.77	-3.75	**0.001**	0.641										
TC1700009528.hg.1	*CXCL16*	58191	9.47	7.42	-2.05	**0.016**	0.708										
TC0300008563.hg.1	*DIRC2*	84925	12.70	10.02	-2.68	**0.034**	0.720										
TC1100007729.hg.1	*DTX4*	23220	10.37	7.84	-2.53	**0.002**	0.646										
TC1200009131.hg.1	*DYNLL1*	8655	7.14	9.61	2.46	**0.024**	0.720			X							X
TC0700011584.hg.1	*FGL2*	10875	7.40	9.59	2.19	**0.041**	0.725										
TC0700010075.hg.1	*GNA12*	2768	13.22	10.98	-2.24	**0.020**	0.708										
TC2200007413.hg.1	*GRAP2*	9402	7.09	9.42	2.33	**0.034**	0.720	X	X			X					
TC1200009221.hg.1	*HIP1R*	9026	10.14	7.77	-2.36	**0.015**	0.708				X			X			
TC2100006433.hg.1	*ICOSLG*	23308	10.22	7.79	-2.43	**0.034**	0.720					X					
TC0100013293.hg.1	*ID3*	3399	10.52	7.80	-2.71	**0.033**	0.720										
TC0400012606.hg.1	*IRF2*	3660	9.82	11.89	2.07	**0.040**	0.721						X		X	X	X
TC1700006772.hg.1	*KDM6B*	23135	12.09	9.90	-2.20	**0.016**	0.708										
TC0700007610.hg.1	*LANCL2*	55915	10.40	7.86	-2.54	**0.038**	0.720										
TC0700013633.hg.1	*LOC154761*	154761	8.96	6.70	-2.27	**0.012**	0.708										
TC0600013726.hg.1	*MIR3918*	100500851	7.30	5.20	-2.10	**0.038**	0.720										
TC0300008605.hg.1	*MIR544B*	100422864	6.85	4.18	-2.67	**0.025**	0.720										
TC1900010502.hg.1	*NFKBID*	84807	11.47	7.21	-4.25	**0.029**	0.720										
TC1200012708.hg.1	*OAS1*	4938	8.69	11.43	2.74	**0.036**	0.720						X		X		
TC0400012938.hg.1	*RASGEF1B*	153020	13.63	10.99	-2.64	**0.040**	0.722										
TC1900008300.hg.1	*RELB*	5971	9.65	7.50	-2.15	**0.034**	0.720										
TC1100009864.hg.1	*RHOG*	391	11.87	9.77	-2.10	**0.049**	0.727										
TC1200010901.hg.1	*RNF41*	10193	11.78	9.70	-2.08	**0.043**	0.727										
TC1200008873.hg.1	*SH2B3*	10019	10.42	7.93	-2.49	**0.026**	0.720	X	X							X	
TC1700012286.hg.1	*SMG8*	55181	8.37	6.29	-2.09	**0.014**	0.708										
TC0600014196.hg.1	*SNX9*	51429	14.56	12.39	-2.17	**0.015**	0.708				X			X			
TC0100009658.hg.1	*SRGAP2C*	653464	10.04	12.19	2.15	**0.041**	0.725										
TC0700009508.hg.1	*TCAF2*	285966	7.85	5.79	-2.06	**0.021**	0.708										
TC0700009511.hg.1	*TCAF2P1*	653691	8.05	5.90	-2.15	**0.039**	0.720										
TC0900008202.hg.1	*TGFBR1*	7046	11.76	9.58	-2.18	**0.009**	0.708										
TC0400010449.hg.1	*TLR10*	81793	11.85	14.56	2.71	**0.012**	0.708										
TC1600008153.hg.1	*TMEM208*	29100	11.31	9.20	-2.11	**0.022**	0.720										
TC0900009827.hg.1	*TOPORS*	10210	8.92	6.81	-2.11	**0.034**	0.720										
TC1700007387.hg.1	*TRAF4*	9618	8.22	5.63	-2.59	**0.031**	0.720										
TC0600011773.hg.1	*TREML2*	79865	6.62	8.98	2.36	**0.002**	0.646										
TC0100009889.hg.1	*VPS45*	11311	9.16	11.29	2.13	**0.049**	0.727									X	
TC0900010019.hg.1	*ZBTB5*	9925	8.93	6.61	-2.32	**0.011**	0.708										
TC0X00009855.hg.1	*ZXDA*	7789	9.26	6.78	-2.48	**0.016**	0.708										

B-cell transcriptome profiles were measured in patients with multiple sclerosis (n=3) before (t0) and after (t1) the treatment of an acute relapse with apheresis. The data were analyzed using paired t-tests to identify upregulated (n=10) and downregulated (n=32) genes with $|log_2FC|>2.0$. The differentially expressed genes are listed in alphabetical order. P-values <0.05 are indicated in bold. In addition to the transcript cluster (probe set) IDs for Clariom D arrays, the NCBI Gene IDs and the top 10 Reactome pathway associations (in the same order as in Figure 4B) are provided.

FC = fold change, FDR = false discovery rate, ID = identifier, NCBI = National Center for Biotechnology Information

Supplemental Table 6 : Gene expression changes in peripheral blood CD4^{+} T cells in response to therapeutic apheresis.

Transcript cluster	Gene symbol	NCBI Gene ID	Mean t0	Mean t1	log_2FC	*p*-value	FDR	R-HSA-162594	R-HSA-982772	R-HSA-380108	R-HSA-909733	R-HSA-1169410	R-HSA-877300	R-HSA-198933	R-HSA-913531	R-HSA-373076	R-HSA-500792
TC0600007203.hg.1	*ACOT13*	55856	6.72	9.00	2.28	**0.024**	0.416										
TC2200009272.hg.1	*APOBEC3D*	140564	9.57	11.88	2.31	**0.048**	0.435										
TC2200009274.hg.1	*APOBEC3G*	60489	8.99	11.03	2.04	**<0.001**	0.266										
TC0200008507.hg.1	*ARID5A*	10865	11.37	9.26	-2.11	**0.023**	0.416										
TC0100008564.hg.1	*ATG4C*	84938	6.02	8.15	2.13	**0.026**	0.416										
TC1100008049.hg.1	*BANF1*	8815	8.53	10.82	2.29	**0.030**	0.416	X									
TC1100008664.hg.1	*C11orf73*	51501	9.02	11.42	2.40	**0.031**	0.416										
TC1100009233.hg.1	*CCDC84*	338657	8.79	6.65	-2.14	**0.020**	0.411										
TC0300007257.hg.1	*CCR5*	1234	6.26	8.67	2.40	**0.035**	0.424	X		X						X	X
TC0300012007.hg.1	*CD200R1*	131450	7.96	10.32	2.36	**0.024**	0.416							X			
TC0X00007132.hg.1	*CDK16*	5127	11.64	9.62	-2.03	**0.008**	0.390										
TC1200010977.hg.1	*CDK4*	1019	6.92	8.98	2.06	**0.029**	0.416										
TC0500011554.hg.1	*CHD1*	1105	16.48	13.73	-2.75	**0.015**	0.401										
TC0300011124.hg.1	*CISH*	1154	6.25	10.97	4.72	**0.023**	0.416		X								
TC1000007272.hg.1	*CREM*	1390	11.75	9.25	-2.50	**0.049**	0.435										
TC0300010775.hg.1	*CX3CR1*	1524	7.43	11.23	3.80	**0.034**	0.424			X						X	X
TC1700009528.hg.1	*CXCL16*	58191	7.78	5.19	-2.60	**0.003**	0.390			X						X	X
TC0900009825.hg.1	*DDX58*	23586	8.51	10.76	2.25	**0.004**	0.390					X			X		
TC0100015265.hg.1	*DENND2D*	79961	11.49	13.79	2.30	**0.020**	0.411										
TC1100009744.hg.1	*DUSP8*	1850	6.59	4.07	-2.52	**0.005**	0.390										
TC1200009131.hg.1	*DYNLL1*	8655	6.96	10.38	3.42	**0.007**	0.390										
TC0600011943.hg.1	*ENPP5*	59084	4.34	6.56	2.23	**0.038**	0.427										
TC0200007096.hg.1	*FOSL2*	2355	10.16	8.06	-2.10	**0.026**	0.416										
TC1200006787.hg.1	*GABARAPL1*	23710	13.88	11.48	-2.40	**<0.001**	0.266										
TC1500007180.hg.1	*GALK2*	2585	7.79	9.98	2.20	**0.042**	0.429										
TC0700009677.hg.1	*GIMAP4*	55303	10.31	13.54	3.23	**0.010**	0.390										
TC0100015747.hg.1	*GOLPH3L*	55204	6.38	9.24	2.85	**0.048**	0.435										
TC0300012812.hg.1	*GPR171*	29909	9.76	12.52	2.76	**0.014**	0.399										
TC1200007652.hg.1	*GRASP*	160622	10.90	8.20	-2.70	**0.003**	0.390										
TC1100009988.hg.1	*GVINP1*	387751	9.59	12.69	3.09	**0.020**	0.411										
TC0200013380.hg.1	*IGK, IGKV1-6,..*	50802	13.38	15.50	2.12	**0.037**	0.427										
TC0400012606.hg.1	*IRF2*	3660	9.66	12.17	2.51	**0.033**	0.422				X		X		X		
TC1300009765.hg.1	*IRS2*	8660	11.07	8.39	-2.68	**0.010**	0.390		X								
TC1200006738.hg.1	*KLRG1*	10219	12.09	14.27	2.18	**0.007**	0.390							X			
TC1900011414.hg.1	*LAIR1*	3903	7.71	9.85	2.14	**0.031**	0.416							X			
TC1200010655.hg.1	*LIMA1*	51474	7.37	9.39	2.02	**0.049**	0.435										
TC0200010337.hg.1	*LOC101927482*	101927482	4.25	6.26	2.02	**0.006**	0.390										
TC1300008892.hg.1	*LPAR6*	10161	11.38	13.42	2.04	**0.017**	0.401									X	X
TC0700012115.hg.1	*NAPEPLD*	222236	6.60	8.73	2.13	**0.003**	0.390										
TC0800011472.hg.1	*NUDCD1*	84955	7.74	9.96	2.22	**0.008**	0.390										
TC1200012708.hg.1	*OAS1*	4938	7.41	10.28	2.88	**0.001**	0.354				X	X	X		X		
TC1200008921.hg.1	*OAS2*	4939	9.83	11.96	2.12	**0.003**	0.390				X	X	X		X		
TC1200007461.hg.1	*PCED1B*	91523	11.57	13.64	2.08	**0.018**	0.406										
TC1600007193.hg.1	*POLR3E*	55718	11.56	9.29	-2.27	**0.008**	0.390										
TC2000007336.hg.1	*PPP1R16B*	26051	12.61	10.43	-2.18	**0.050**	0.436										
TC1400007165.hg.1	*PTGER2*	5732	9.25	12.22	2.97	**0.021**	0.413									X	X
TC0200007015.hg.1	*RAB10*	10890	10.05	12.67	2.62	**0.011**	0.390										
TC1100011797.hg.1	*RAB30*	27314	11.26	8.75	-2.51	**0.044**	0.430										
TC0700008072.hg.1	*RHBDD2*	57414	11.51	9.10	-2.41	**0.012**	0.390										
TC0200016704.hg.1	*RNF103*	7844	12.82	10.65	-2.17	**0.011**	0.390										
TC0100007567.hg.1	*RNU11*	26824	9.99	12.49	2.50	**0.012**	0.390										
TC1200012156.hg.1	*RNU4-1*	26835	13.81	16.08	2.27	**0.009**	0.390										
TC1200012155.hg.1	*RNU4-2*	26834	15.65	17.78	2.13	**0.029**	0.416										
TC0100015822.hg.1	*S100A11*	6282	11.46	14.69	3.23	**0.011**	0.390										
TC0700011797.hg.1	*SAMD9L*	219285	8.98	12.50	3.52	**0.010**	0.390										
TC0100016135.hg.1	*SLAMF6*	114836	12.73	16.34	3.60	**0.021**	0.411							X			
TC0300008989.hg.1	*SLC25A36*	55186	12.24	10.13	-2.11	**0.015**	0.401										
TC0300009789.hg.1	*ST6GAL1*	6480	10.26	13.43	3.17	**0.042**	0.429										
TC0700009511.hg.1	*TCAF2P1*	653691	10.59	8.45	-2.14	**0.020**	0.411										
TC0600013125.hg.1	*THEMIS*	387357	12.83	15.26	2.43	**0.035**	0.424										
TC1500010731.hg.1	*TRIM69*	100419583	6.60	8.71	2.11	**0.034**	0.423										
TC0100016685.hg.1	*TRMT1L*	81627	5.84	8.21	2.37	**0.038**	0.427										
TC0400011383.hg.1	*TSPAN5*	10098	7.26	9.39	2.13	**0.004**	0.390										
TC1900008329.hg.1	*VASP*	7408	8.54	10.73	2.18	**0.039**	0.427										
TC1500008100.hg.1	*WHAMM*	123720	13.98	11.71	-2.27	**0.030**	0.416										
TC0300010350.hg.1	*WNT7A*	7476	10.36	7.85	-2.51	**0.005**	0.390										X
TC0600011162.hg.1	*ZNF322*	79692	4.92	7.18	2.26	**0.030**	0.416										
TC0100008996.hg.1	*ZNF326*	284695	12.56	10.54	-2.01	**0.007**	0.390										
TC1900008755.hg.1	*ZNF331*	55422	13.73	10.72	-3.01	**<0.001**	0.266										

Transcriptome profiles of $CD4^+$ T cells were measured in patients with multiple sclerosis (n=4) before (t0) and after (t1) relapse treatment with apheresis. The data were analyzed using paired t-tests to identify upregulated (n=47) and downregulated (n=22) genes with $|\log_2 FC|>2.0$. The differentially expressed genes are listed in alphabetical order. P-values <0.05 are indicated in bold. In addition to the transcript cluster (probe set) IDs for Clariom D arrays, the NCBI Gene IDs and the top 10 Reactome pathway associations (in the same order as in Figure 4B) are provided. Of note, *GRASP* and *ZNF331* were found to be upregulated in response to methylprednisolone therapy ($\log_2 FC>2.0$) in our previous study [Hecker et al., Biomed Pharmacother, 2024].

FC = fold change, FDR = false discovery rate, ID = identifier, NCBI = National Center for Biotechnology Information
